# Barriers and facilitators to implementation of oral rehydration therapy in low- and middle-income countries: A systematic review

**DOI:** 10.1371/journal.pone.0249638

**Published:** 2021-04-22

**Authors:** Obidimma Ezezika, Apira Ragunathan, Yasmine El-Bakri, Kathryn Barrett

**Affiliations:** 1 Department of Health and Society, University of Toronto Scarborough, Toronto, Canada; 2 Dalla Lana School of Public Health, University of Toronto, Toronto, Canada; 3 Faculty of Health Sciences, Ontario Tech University, Oshawa, Canada; 4 African Centre for Innovation and Leadership Development, Abuja, Nigeria; 5 University of Toronto Scarborough Library, Toronto, Canada; Universidad de Antioquia, COLOMBIA

## Abstract

**Background:**

Oral rehydration therapy (ORT) is an effective and cheap treatment for diarrheal disease; globally, one of the leading causes of death in children under five. The World Health Organization launched a global campaign to improve ORT coverage in 1978, with activities such as educational campaigns, training health workers and the creation of designate programming. Despite these efforts, ORT coverage remains relatively low. The objective of this systematic review is to identify the barriers and facilitators to the implementation of oral rehydration therapy in low and middle-income countries.

**Methods:**

A comprehensive search strategy comprised of relevant subject headings and keywords was executed in 5 databases including OVID Medline, OVID Embase, OVID HealthStar, Web of Science and Scopus. Eligible studies underwent quality assessment, and a directed content analysis approach to data extraction was conducted and aligned to the Consolidated Framework for Implementation Research (CFIR) to facilitate narrative synthesis.

**Results:**

The search identified 1570 citations and following removal of duplicates as well as screening according to our inclusion/exclusion criteria, 55 articles were eligible for inclusion in the review. Twenty-three countries were represented in this review, with India, Bangladesh, Egypt, Nigeria, and South Africa having the most representation of available studies. Study dates ranged from 1981 to 2020. Overarching thematic areas spanning the barriers and facilitators that were identified included: availability and accessibility, knowledge, partnership engagement, and design and acceptability.

**Conclusion:**

A systematic review of studies on implementation of ORT in low- and middle-income countries (LMICs) highlights key activities that facilitate the development of successful implementation that include: (1) availability and accessibility of ORT, (2) awareness and education among communities, (3) strong partnership engagement strategies, and (4) adaptable design to enhance acceptability. The barriers and facilitators identified under the CIFR domains can be used to build knowledge on how to adapt ORT to national and local settings and contribute to a better understanding on the implementation and use of ORT in LMICs. The prospects for scaling and sustaining ORT (after years of low use) will increase if implementation research informs local applications, and implementers engage appropriate stakeholders and test assumptions around localized theories of change from interventions to expected outcomes.

**Registration:**

A protocol for this systematic review was developed and uploaded onto the PROSPERO international prospective register of systematic reviews database (Registration number: CRD420201695).

## Introduction

Diarrheal disease remains one of the leading causes of death globally [1]. In high-income countries, mortality rates have decreased significantly over the past few decades, however mortality remains high in low and middle-income countries [1]. Countries in the regions of Africa, South America and Asia that have the lowest GDP hold the highest mortality rates among children under five due to diarrheal disease [2].

In 1968, glucose-electrolyte solutions were found to successfully treat cholera in patients [3]. This solution is described as oral rehydration solution (ORS), and is a type of oral rehydration therapy (ORT) used commonly to treat dehydration [4]. Today, ORS is described as any packaged rehydration solution containing a form of sugar, together with additional electrolytes, such as sodium or potassium. An alternative form of ORT also exists, described as Recommended Home Fluids (RHF). RHF includes any non-packaged home fluid alternative ORT treatment such as cereal-salt, rice-water or sugar-salt solutions, as well as other common home fluids such as juice or tea [4]. Since 1978, ORT has been widely implemented in many countries by the World Health Organization to treat diarrheal diseases [5]. For this review, ORT was defined as any rehydration solution used to treat diarrhea in children under five years of age. This definition allowed for the encompassing of any variety of rehydration solutions mentioned within included articles in our results. The two most common types of oral rehydration therapies addressed in the articles were oral rehydration solutions (ORS) and recommended homemade fluids (RHF).

Through a rigorous WHO campaign, ORS has been used to treat millions of cases of diarrheal disease. Since 2007, it has helped save almost 54 million lives [6]. Despite its success in reducing global mortality, coverage still remains low today. In the 1990s, ORT and diarrheal program promotion and planning had stagnated, and the focus was shifted from diarrheal control to treating all childhood illnesses [7]. This negatively affected ORS coverage, which is currently only at 43% globally, meaning that only less than half of people in need of it are receiving it [8]. Consequently, in 2017, almost 300,000 diarrheal deaths among children in low- and middle-income countries (LMICs) could have been prevented had ORS been administered [4]. A recent examination on the progress of ORS implementation summarized some of the reasons as to why the progress on ORS coverage has stagnated over the past few decades and espoused for a more multipronged approach to increasing ORS coverage [9]. These reasons include lack of political commitment, insufficient resources and infrastructure, and/or socio-cultural factors such as the lack of perceived benefit of ORS at the community level [10], which have translated to a lack of ORS supplies, poor distribution of ORS, inadequate government level policies regarding ORS and its distribution, low awareness of ORS usage and poor training of health workers [9].

There needs to be an increase in coverage and uptake of ORT in countries facing the heaviest mortality and morbidity rates. This would help avoid preventable deaths of children under-five. Typically, children with diarrhea experience fluid loss and dehydration, which often results in death if left untreated [11]. This fluid loss can be easily addressed through the administration of ORT, a cost-effective and simple treatment which can relieve dehydration in affected individuals and decrease diarrhea related mortality [12]. Be that as it may, ORT coverage is inconsistent across LMICs, with high coverage observed in South and East Asia, Central America and Southern Sub-Saharan Africa, and low coverage in Central, West and East Sub-Saharan Africa, North African, Middle East and South America [4].

Aside from the need for increased diarrheal program promotion and planning, there is still a lack of understanding and a structured organization of the underlying factors to ORT implementation and usage in LMICs. While systematic and narrative reviews related to ORT exist and have explored issues pertaining to coverage and uptake of ORT [10, 13, 14], these reviews have not addressed facets related to implementation science. These reviews have either examined the effectiveness of ORT in addressing mortality rates from deaths from childhood pneumonia and diarrhea [13], strategies to increase uptake of ORS [10], or country cases on why ORS failed or succeeded [14]. There have also been critical commentaries on ORT implementation [11, 15]. However, there has been no systematic review conducted on the barriers and facilitators to ORS and RHF implementation, organized according to a determinant implementation science framework.

To our knowledge, this is the first systematic review of the barriers and facilitators that exist in the implementation of ORT in low and middle-income countries. The hope of this study is that these findings will provide a more structured organization of the barriers and facilitators to ORT implementation and help support the generation of hypotheses for improving the implementation of ORT in LMICs.

## Methods

This systematic review is presented according to the Preferred Reporting Items for Systematic Reviews and Meta-Analysis (PRISMA) 2009 checklist [16]. The protocol that was developed for this study [17] has since been uploaded onto the PROSPERO database according to standard practice in systematic reviews.

### Eligibility

Eligibility criteria were selected to examine studies that would capture data relevant to implementation science and ORT in LMICs (Table 1). Eligible studies focused on the implementation of ORT, getting evidence into practice, the scale-up of ORT, and/or methods and tools that allowed implementation of ORT. These studies’ objectives focused exclusively on either ORT practices, diarrheal management involving ORT practices or ORT implementation with zinc. We included studies addressing populations who have used and participated in ORT practices. These were populations aged 5 years or younger, as they are the main population affected by the disease (diarrhea), and their caregivers. Stakeholders involved in the implementation of ORT for children under five were also included, such as researchers, policy makers, or healthcare professionals. Included studies were in the English language, published in a scholarly journal and followed mixed-methods, qualitative, or quantitative methodology.

**Table 1:** Summary of inclusion and exclusion criteria.

Selection Criteria	Inclusion Criteria	Exclusion Criteria
*Publication Characteristics*		
**Language**	English	All languages except English
**Publication Type**	Research articles published in a scholarly journal	Review articles published in a scholarly journal
		All publications that are not scholarly journal articles
**Publication Date**	1960–2020	Articles published before 1960
**Study design**	Qualitative (e.g. focus group discussions, interviews) quantitative (e.g. structured/semi-structured questionnaires, household surveys, etc.), randomized control trials, non-randomized trials or mixed-methods research studies focusing on implementation and scale-up of ORT	Research articles with no methodology listed, or with a focus other than the implementation and scale-up of ORT
*Study Characteristics*		
**Issue**	Diarrheal disease	All other diseases
**Population**	Children under 5 years of age	Children over 5 years of age
	Caregivers of children under 5 years of age	Adolescents, adults, and seniors who are not caregivers of children under 5 or who are not involved in the implementation of oral rehydration therapy
	Stakeholders involved in the implementation of oral rehydration therapy targeted at children under 5 years of age	
**Location**	Low- and middle-income countries, based on World Bank criteria	High-income countries, based on World Bank criteria
**Intervention**	Oral rehydration therapy (ORT), including diarrheal management involving ORT practices or ORT with zinc	Oral rehydration therapy (ORT) when implemented with non-related practices (e.g. vaccines, antibiotics)
		Diarrheal management with zinc only
**Outcome**	Discussion of the barriers and facilitators to the implementation or scaling up of oral rehydration therapy interventions	No discussion of the barriers and facilitators to implementation or scaling up of oral rehydration therapy interventions

Studies discussing implementation of non-related practices along with ORT, such as vaccines or antibiotics, were excluded. We excluded articles focusing on implementation of ORT for populations older than 5 years of age or not exclusively focused on ORT, such as programs where ORT is only an aspect or zinc is the sole focus for diarrhea treatment. Articles that did not focus on countries in low and middle-income countries, as based on the World Bank database of low and middle-income countries [18] were also excluded. Examples of studies that were excluded because of ineligibility were case studies with no methodology listed, or studies from journals that are not peer reviewed.

### Search strategy

The search was performed in five databases, EMBASE (OVID), HealthSTAR (OVID), Medline (OVID), Web of Science, and Scopus. This search was executed in all five databases on February 27th, 2020. Scholarly journal articles were included, with grey literature being excluded. Each search string included key terms that were based on the four main concepts of our research question, which were oral rehydration therapy, implementation science, diarrhea treatment, and the population of children under five years old. Subject headings and keywords were used in Medline, HealthStar and Embase. Keywords were used in Web of Science and Scopus. Specific search strategies used for each database can be found in S1 File.

### Screening strategy

**Fig 1:**
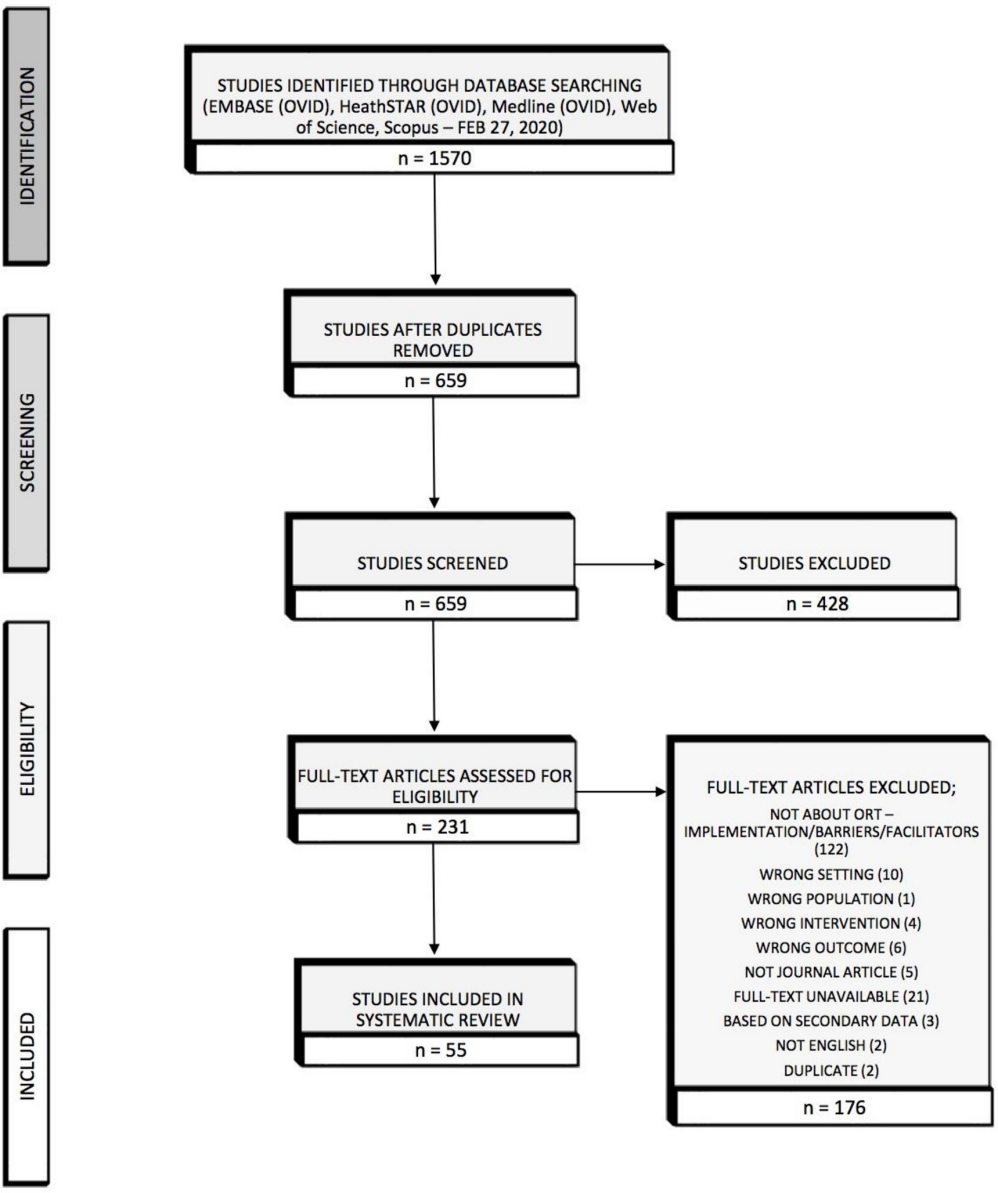
PRISMA diagram.

Studies went through a four-step process to identify which ones would be included. The first stage was identifying all articles that were retrieved through the five databases (n = 1570) and uploading them into Covidence, a systematic review management tool for screening and data extraction. The next stage was to remove all duplicates found across databases to create a list of studies to screen based on our criteria (n = 659). Studies were screened in two rounds using Covidence. During the first round, titles and abstracts were screened by two authors. Studies were excluded if the title and/or abstract did not meet all the eligibility criteria. If insufficient information was present to make a conclusion, the study was moved to the second stage of screening for further review. A third author resolved any disagreements on article eligibility. A total of 428 articles were excluded during this first round. During the second round of screening, the initial two authors independently performed full-text screening on the remaining 231 articles and the third author resolved any discrepancies. Studies were excluded if the full text did not meet all of the eligibility criteria (Table 1). Reasons for studies exclusion at this stage included: not being about ORT implementation or no extractable barriers or facilitators (n = 122), wrong setting (n = 10), wrong population (n = 1), wrong intervention (n = 4), wrong outcome (n = 6), not a scholarly journal article (n = 5), full-text unavailable (n = 21), conclusions based on secondary data (n = 3), not in English (n = 2), and duplicates (n = 2). In total, 177 articles were excluded at the second round of screening. After screening, 55 articles moved forward to data extraction. The Preferred Reporting Items for Systematic Reviews and Meta-Analysis (PRISMA) framework was used to report our systematic review (See S2 File), and each stage of our screening process can be found in our PRISMA diagram (Fig 1).

#### Quality assessment

Studies that were selected for data extraction were assessed using the Mixed Methods Appraisal Tool (MMAT), a critical appraisal tool developed for systematic reviews that include qualitative, quantitative, and mixed methods studies. The MMAT was selected, as our study covered qualitative studies, quantitative descriptive studies, quantitative randomized control trials, quantitative non-randomized studies and mixed methods studies. The MMAT is comprised of a list of criteria to appraise the methodological quality of five types of studies, scoring them on a scale ranging from 0, a low quality study, to 4, a high quality study [19]. A further description of the tool and the results of this application can be found in S1 Table, which includes the scores from 0–4 of all 55 of our studies to identify them as low- or high-quality studies using the MMAT, as well as the questions used to appraise the quality of the study to which the scores are addressed. There were two rounds of questions applied to each study. The first round included two screening questions regarding the research question and the data collected. During the second round of questions, each study was assigned to 1 of 5 study types (qualitative studies, quantitative randomized control trials, quantitative non-randomized studies, quantitative descriptive studies, and mixed-methods studies), and there were five unique questions that were answered for each particular study type. For example, quantitative studies were appraised based on the five questions related to quantitative studies. Each author appraised the studies separately, then came together to reach consensus.

#### Data extraction

Two of the authors extracted the following data items from each study: study title, year of publication, author names, country of study, and study design/methods (S2 Table). In addition, both the barriers and facilitators to ORT implementation were extracted from each article. The two authors were blinded and independently extracted relevant data from each article in separate documents to ensure rigor. A third author was brought in to resolve any conflicts between the two documents.

#### CFIR application

The Consolidated Framework for Implementation Research (CFIR) is a conceptual framework created to guide the systematic assessment of factors that influence the implementation and effectiveness of interventions [20]. Incorporating CFIR during the analysis and synthesis phase of this review was extremely beneficial, as integrating a conceptual framework increased both the generalizability and interpretability of study results. The CFIR codebook is quite comprehensive and describes 5 broad domains and 39 constructs including construct definitions and examples [20]. The CFIR was helpful in triangulating information and also allowed us to conceptualize the barriers and facilitators in a more organized manner. Application of the CFIR was assessed by reviewing each barrier and facilitator that was extracted and coding them according to one of the 39 constructs of the CFIR framework.

Two authors independently coded each facilitator and barrier according to the CFIR framework and then came together to identify and resolve any disagreements between the two sets of data that was coded. The third author was brought in to further resolve any remaining conflicts that the first two authors may have had when coding the collected data, by determining which CFIR code for each barrier and facilitator would be most appropriate based on the CFIR definitions. A consolidated document containing all relevant barriers and enablers extracted from included articles with the associated CFIR constructs was developed (S3 and S4 Tables).

## Results

From the OVID Medline, OVID Embase, OVID HealthSTAR, Web of Science, and Scopus databases, we screened 1570 studies references and assessed the full text of 659 documents. Fifty-five publications met our inclusion criteria, and their data was extracted. Authors reached consensus on eligibility of all the included studies. Table 1 summarizes the fifty-five studies, including important study elements, study design methods, participants, and study objectives.

### Type of study/methods

The 55 articles included in our study used a combination of several methods, including various quantitative, qualitative, and mixed methods. Out of the 55 articles included in our study, 36 were interventional studies and 19 were non-interventional studies. An interventional study is one where the researchers intervene in the study to collect data on a particular outcome, such as introducing a new program to a population. A non-interventional study is where researchers collect data on relationships between an outcome and a factor that exist in a setting without implementing an intervention, such as looking at knowledge patterns amongst a population [21].

The 36 interventional studies in our study used a variety of study methods. Most interventional studies were quantitative (n = 14), followed by qualitative studies (n = 9), and lastly mixed methods (n = 4). Randomized control trials (n = 5) and non-randomized control trials (n = 4) were only present in the interventional studies. Each study type employed different data collection methodology, either using one type of method (such as using a survey) or a combination of several (such as using a survey and interviews). Surveys were used most frequently in interventional studies (n = 21), either alone or in combination with other methods. They were especially employed in the quantitative studies (n = 9). Many interventional studies also used the analysis of secondary data, such as government statistics and hospital records (n = 12), in combination with other methods. Methods of monitoring, observation, evaluation, and surveillance were also used mostly in quantitative studies (n = 5) and qualitative studies (n = 3). Interviews were used as the most common method in qualitative studies (n = 6), but interviews were not used at all in non-randomized control trials. Focus groups were the least common method used across all studies (n = 5), with the randomized control trials and non-randomized control trials not using them at all.

There were 19 non-interventional studies, in which qualitative (n = 10), quantitative (n = 7) and mixed methods (n = 2) were used. Similar to the interventional studies, interviews were the most common method used in non-interventional qualitative studies (n = 8). Focus groups were the least common method used, as they were employed in one qualitative study (n = 1). Methods of monitoring, observation, surveillance and evaluation were used across all study types (qualitative, quantitative and mixed methods). Surveys were also used across all study types, but most frequently in the quantitative studies (n = 3). Secondary data was used in 15.7% of all non-interventional studies and was present in qualitative and quantitative studies.

### Year of publication

Our search was designed to include studies published between 1960-present. This time period was selected as ORT was initially developed during the 1960s and started to grow in usage in the 1970s [22]. While there were no studies published between 1960–1980, thirty-one studies were published between 1981–2000 and included in our review. Five studies were published between 2001–2010, and nineteen were published between 2011–2020.

### Participants

Study participants ranged from general populations to specific stakeholders involved in ORT implementation. Of the fifty-five studies, 19 studies involved the general population in designated target areas as participants. These general populations were involved in the process of implementing ORT to the target population of children under-five. While other included studies specified a particular population in their methods, such as caregivers and their children under-five, pharmacists, or health workers, the general population was designated to studies that did not have a well-defined population, but instead referred to their population of study as villages, communities, households and other key groups. As mothers, caretakers and females of reproductive age provide primary care and administration of ORT to children, this cohort was observed as participants in 15 studies. Nine studies used children under-five as their study population, and children were either at risk of diarrhea or had diarrhea. Frontline and health workers involved in distribution and treatment of diarrhea, being medical practitioners (n = 2) and pharmacists (n = 1), were also involved. One study included data from health facilities, who were involved in outreach and provision of ORT.

There were 8 studies that studied more than one type of population. These studies expanded on the populations already listed, further including health workers (such as outreach workers) and government workers. Three different studies looked at children under-five using ORT, with 1 of the 3 also looking at health facility providers, and 2 of the 3 looking at physicians. Two studies looked at a general population, with 1 of the 2 studies was focused on ORT providers (village doctors, pharmacists, and government workers), and the other was focused on mothers of children-under five. Two different studies looked at mothers of children under-five, with 1 of the 2 looking at a group of health workers (physicians, traditional healers, and health care providers) and the other looking at a medical practitioners and pharmacists. One study looked at physicians and families as their target population.

### Countries

The fifty-five studies included in this review focused on low and middle-income countries based on World Bank criteria [18]. Of the countries listed by the World Bank, 23 countries were featured in our 55 included studies: Nigeria; India; Myanmar; Philippines; Bangladesh; Kingdom of Tonga; South Africa; Haiti; Ghana; Egypt; Papua New Guinea; Ethiopia; Nicaragua; Mexico; Guatemala; Malawi; Thailand; Burundi; Kenya; Uganda; Indonesia; Pakistan; and Brazil. Countries located in Africa were most frequently present amongst the studies, with 24 of the 55 studies being conducted in Africa. Studies were set most predominately in Egypt (n = 6) and Nigeria (n = 5), followed by South Africa (n = 3). Fewer studies were set in other geographies in Africa, including Ethiopia (n = 2), Kenya (n = 1), Uganda (n = 1), and even less frequently in Ghana (n = 1), Burundi (n = 1) and Malawi (n = 1). South Asian countries were also featured frequently across the 55 studies, with 21 studies being set in South Asia. The most predominant setting in South Asia was India (n = 11), followed by Bangladesh (n = 7), and the least frequent country featured from South Asia was Pakistan (n = 3). Four studies were set in countries of Southeast Asia, specifically in Myanmar (Burma) (n = 1), Philippines (n = 1), Thailand (n = 1), and Indonesia (n = 1). The remaining studies were set in other geographies, with the following countries featured in 8 studies; Mexico (n = 2), Brazil (n = 1), Guatemala (n = 1), Haiti (n = 1), Kingdom of Tonga (n = 1), Nicaragua (n = 1), and Papua New Guinea (n = 1). One study was set in multiple countries including India, Nigeria, Kenya and Uganda (n = 1).

### MMAT

Based on the MMAT, all fifty-five studies met the screening criteria that can be found in S1 Table. Therefore, the studies each presented clear research questions and collected data that addressed these questions. The overall mean quality score for the included studies was 3.74 (high quality). When broken down, 44 of the 55 studies (80.0%) received a score of 4, and 9 of the 55 (16.4%) studies received a score of 3. One study received a score of 2, and another received a score of 1. No studies scored an MMAT rating lower than 1. The types of studies however differed in their mean scores. The nineteen qualitative studies received a mean score of 4, of which 9 were interventional and 10 were non-interventional. The twenty-one quantitative studies received a mean score of 3.81, 14 of which were interventional and 7 of which were non-interventional. The six mixed-methods studies received a mean score of 3.5, 4 of which were interventional and 2 of which were non-interventional. Of the five interventional studies that used randomized control and four interventional studies that used non-randomized methods, the mean scores were 3.2 and 3.25, respectively. The complete list of MMAT scores for all fifty-five studies is presented in S1 Table.

### Study objectives of the selected papers

Fifty-five studies analysed implementation of ORT in different ways. Sixteen studies investigated the awareness and knowledge of ORT from different populations, including mothers, caregivers, communities, and doctors. These studies examined knowledge/awareness of ORT in terms of its purpose and correct preparation, after implementation of training/education programs, collected data on pre-existing knowledge/awareness, or studied the ability to prepare ORT. There were seven studies that investigated the gaps and drivers of implementing diarrheal management and ORT uptake. This included collecting evidence on resources that could improve implementation, and gaps that inhibit implementation, such as supplies, socioeconomic status that affects literacy, and medical practices. This review observed eight studies that examined interventions that sought to increase ORT coverage and overcome barriers such as low availability in rural areas. Four studies evaluated the impact of community engagement, such as developing national programs and mass communication campaigns, the focus of such programs and campaigns being ORT awareness (spreading awareness of ORT, but not the training to prepare ORT) and distribution. There were fourteen studies that explored programs in more local contexts, such as ORT corners in hospitals and community-based ORT programs. Finally, this study observed six studies that evaluated new or alternative designs of ORT, such as rice-based ORT or packaged ORT.

### ORS or RHF

Among the 55 studies included, 40 were exclusively on ORS and 15 studies mentioned RHF as their rehydration therapy of study. Among these articles, 11 of the 15 studies (73.3%) focused solely on RHF, whilst 4 of the 15 studies (26.7%) focused on both RHF and ORS. Specifically, with regards to those 11 articles which were solely focused on RHF; 4 articles focused on homemade sugar and salt solution (SSS), 2 focused on *lobon-gur* solution (LGS), 1 focused on rice-based oral rehydration solution (R-ORS), 1 focused on homemade cereal based oral rehydration therapy (HC-ORT), and 2 focused on non-specified homemade ORS. One study looked at both homemade cereal-based ORS and rice-based ORS.

### Barriers and facilitators

After reviewing the consolidated document containing all relevant barriers and facilitators extracted from included articles, researchers noted four themes which naturally emerged from the data related to availability and accessibility, knowledge, partnership engagement, and design and acceptability. To provide the reader with a sense of the diverse contexts across studies, sample quotes from articles representative of the meta-themes associated with CFIR domains and constructs were listed (S3 and S4 Tables). In addition, the frequency for each construct of the CFIR is summarized in Table 2.

**Table 2:** Frequency table of cited Consolidated Framework for Implementation Research (CFIR) constructs (N = 55).

CFIR domains (n = 5) and constructs (n = 39)	Barrier n (%) of studies	Facilitator (%) of studies
I. Intervention characteristics		
*No facilitators or barriers were noted for these constructs related to Intervention Source*, *Evidence Strength and Quality*, *Relative Advantage*, *Trialability*, *Complexity*		
Adaptability	None Identified	4 (7.2%)
Design Quality and Packaging	2 (3.6%)	3 (5.4%)
Cost	3 (5.4%)	None Identified
II. Outer Setting		
*No facilitators or barriers were noted for these constructs related to Patient Needs and Resources*, *Peer Pressure*		
Cosmopolitanism	None Identified	3 (5.4%)
External Policy and Incentives	None Identified	4 (7.2%)
III. Inner Setting		
*No facilitators or barriers were noted for these constructs related to Structural Characteristics*, *Network and Communications*, *Culture*, *Tension for Change*,^*1*^* Compatibility*,^*1*^* Relative Priority*,^*1*^* Organizational Incentives and Rewards*,^*1*^ *Goals and Feedback*,^*1*^* Learning Climate*, ^*1*^ *Leadership Engagement*^*2*^		
Available Resources—Readiness for Implementation	6 (10.9%)	2 (3.6%)
Access to Knowledge and Information—Readiness for Implementation	9 (16.3%)	8 (14.5%)
IV. Characteristics of Individuals		
*No facilitators or barriers were noted for these constructs related to Self-Efficacy*, *Individual Stage of Change*, *Individual Identification with Organization*		
Knowledge and Beliefs about the Intervention	15 (27.2%)	None Identified
Other Personal Attributes	2 (3.6%)	None Identified
V. Process		
*No facilitators or barriers were noted for these constructs related to Planning*, *Opinion Leaders*, ^*3*^ *Formally Appointed Internal Implementation Leaders*, ^*3*^ *Champions*,^*3*^*External Change Agents*, ^*3*^ *Executing*, *Reflecting and Evaluating*		
Engaging	None Identified	8 (14.5%)

^1^sub-constructs of Implementation Climate.

^2^sub-constructs of Readiness for Implementation.

^3^sub-constructs of Engaging.

The CFIR was useful in orienting researchers to implementation factors, and provided increased rigor for data extraction. The grouping of barriers and facilitators into broader themes using the CFIR assisted researchers in imposing logic and creating sense from a previously overwhelming list of constructs. In addition, of the several constructs available under the CFIR, 26% were used in this review, and further grouped under four newly developed major themes. The grouping under these themes can assist in further understanding which factors and constructs are relevant in the context of future ORT implementation. Below, we present the four major themes with the relevant CFIR domains and constructs addressed within each theme: availability and accessibility, knowledge, partnership engagement, and design and acceptability (Table 3).

**Table 3:** Thematic table of cited Consolidated Framework for Implementation Research (CFIR) constructs.

Themes	Description	CFIR construct (CFIR domain)
**Availability and Accessibility**	*The availability of ORT in the context of supply and demand and its accessibility in the terms of cost and location*.	◾ Available Resources (Inner Setting)
		◾ Cost (Intervention Characteristics)
**Knowledge**	*Community’s knowledge and awareness on what ORT is*, *attitudes towards ORT*, *literacy and understanding of diarrhea/ORT*, *how health workers are trained and educated*, *and the existence of ORT education and promotion*	◾ Knowledge and Beliefs about the Intervention (Characteristics of Individuals)
		◾ Access to Knowledge and Information (Inner Setting)

**Partnership Engagement**	*External entities (government*, *community programs*, *private sector*, *etc*.*) that work to increase ORT uptake amongst population*. *This includes subsidizing resources*, *running communication campaigns*, *creating partnerships and developing programs*.	◾ Engaging (Process)
		◾ Cosmopolitanism (Outer Setting)
		◾ External Policies and Incentives (Outer Setting)
**Design and acceptability**	*The design and quality of the innovation and how it affects acceptability and uptake of the innovation among various settings/cultures*. *This includes the existence of other treatments*, *more culturally adapted designs of ORT*, *acceptance amongst communities and taking cultural norms into account*.	◾ Design and Quality and Packaging (Intervention Characteristics)
		◾ Other Personal Attributes (Characteristics of Individuals)
		◾ Adaptability (Intervention Characteristics)

#### Availability and accessibility

*Available resources (inner setting)*. The availability and accessibility of ORT was commonly cited as a factor influencing its implementation, with 8 of the 55 studies citing the low supply or low access to ORT as a barrier. Of these studies, four mentioned how implementation and scale-up efforts were limited by low availability of ORS and related materials in Bangladesh, Nigeria, Egypt and India [23–26]. Research in Egypt found that “almost 100,000 preschool children died annually from diarrheal disease in rural Egypt alone. One third to one half of these deaths could have been prevented with an effective ORT programme; yet the main obstacle to the implementation of such a programme was the lack of ORS” [25] (p198), underscoring the consequences of low availability of ORT. Additionally, in two of the eight studies, residents of rural areas presented the lowest uptake not only due to limited access to supplies, but also because of limited access to information on ORT [27], and general shortages of cash for purchasing ORS packets [28]. Problems with distribution [29] and commercial production of packets [23] were also cited in two studies set in the Philippines and Bangladesh, as barriers to implementation, which further emphasized the limited supply of ORS [23, 29]. Research in the Philippines identified that “although ORS production was sufficient, there were problems in its distribution, which led to its underuse. ORS was unavailable in some areas and in excess supply in others” [29] (p638), highlighting our review’s findings on the impact of ORS distribution and supply to implementation.

However, it is important to note that in one study the regular availability of ORS was highlighted as a facilitator to uptake and implementation [30], which is unsurprising given the results of 5 of the 8 studies presented above, which mentioned low availability as a barrier to implementation [23–26, 29]. In addition, the implementation of a social franchising program in Myanmar was cited by one study as a facilitator, with diarrhea treatment and uptake of ORS and zinc increasing significantly in intervention populations after implementation of the program [31].

*Cost (intervention characteristics)*. Of the 55 studies, 3 studies cited the high cost of ORT as a barrier, as the cost made it both unavailable and inaccessible to poor populations [27, 28, 32]. Two of the 3 studies, one conducted in Haiti and one in Pakistan, observed that the cost of ORS and ORT respectively was a barrier to implementation [27, 28]. The study that took place in Pakistan found that “the cost of the packets predisposed to underuse, given the cost and difficulty of transport and the general shortage of cash in this rural population” [28] (p56), emphasizing the critical role cost plays in implementation. Cost specifically affected uptake amongst the poor [28], and those living in rural areas who opted for cheaper alternatives if available [27] or were unable to pay for ORT due to a lack of funds [28]. Cost was also preventative in transporting and distributing ORT, which inhibited people from using them [27, 32]. The costs of packaging, production and distribution also affects the availability of ORT in primary health care settings in Bangladesh [32].

#### Knowledge

*Knowledge and beliefs about the intervention (characteristics of individuals)*. Low knowledge and understanding of ORT was largely cited as a barrier to implementation, found in 14 studies [28, 33–45]. In 6 of these 14 studies, low knowledge of ORT amongst users was a barrier [28, 33–37]. A study in north-eastern Nigeria administered a structured questionnaire survey to 518 mothers, and found that “approximately 40 to 80% of the respondents in the survey either had no idea of ORT or have an inappropriate perception of the function of ORT.” [33] (p238), further concluding that the lack of knowledge of the purpose of ORT is an impediment to its use. Low knowledge on ORT preparation also inhibited its uptake and scale-up [33, 38], as incorrect preparation of ORT, which renders the rehydration solution ineffective, lead to misconceptions of the effectiveness of ORT [33]. Two out of the 6 studies that cited inadequate knowledge were published between 2000 and 2019 [34, 37], whilst the remaining 4 were published prior to the year 2000. This suggests that low knowledge and understanding of ORT, although still an obstacle, may not be as relevant of a barrier in the context of implementation, nor as pressing to address when compared to the other more recently studied barriers listed in this review.

Negative attitudes to ORT was cited in 5 of the 55 studies as a barrier to implementation, specifically amongst caregivers [40–44]. Research in Thailand detailed the extent of caregivers’ attitudes towards ORS, “Whilst WHO-ORS was cheaper for public hospitals to obtain, the ambivalent status of ORS as a non-exclusive, low-technology, inexpensive medicine, and the fact that infants were unable or unwilling to swallow the salty-tasting liquid, meant it was not well accepted by either prescribers or caretakers” [42] (p1034), thus contextualizing mothers’ negative attitudes as a barrier to implementation. Low knowledge of diarrhea amongst Nicaraguan mothers providing treatment to their children was also cited as a barrier [45]. Two of the 55 studies, one set in Pakistan and one in Bangladesh, found that culture affected ORT implementation, as locals preferred to use alternative treatments commonly used in their culture [28, 46]. In Bangladesh, drugs and allopathic medicines were commonly used to treat diarrhea, while the local RHF solution called lobon-gur was used in only 4–10% of diarrhea cases [46]. Diarrhea was also defined differently in some cultures, affecting the uptake of ORS as a treatment. In Pakistan, diarrhea was considered a “folk illness”, and locals therefore used traditional folk treatments rather than ORT [28].

*Access to knowledge and information (inner setting)*. Implementation was successful where caregivers were able to access knowledge about diarrhea treatment and ORT [31, 45, 47–52]. However, healthcare professionals were shown to be important influencers in the implementation of ORT, as studies found that lack of promotion and distribution of ORT by doctors [53–56] and pharmacists [57, 58] served as a barrier to implementation. A study in Bangladesh and a study in Egypt found that doctors and pharmacists respectively did not promote ORS as a treatment to diarrhea [54, 57], while four studies found that these healthcare professionals prescribed drugs, including antimicrobials [55], antidiarrheals [55, 56, 58], antiemetics [56], antibiotics [53, 56, 58], and spasmolytics [53], instead of ORT [53, 55, 56, 58]. Three of these four studies were published before the year 2000, further alluding to low knowledge as a barrier not being as relevant in today’s context of ORT implementation. Two studies cited poor education/training of health workers as a barrier to access to knowledge, as poorly educated/trained health workers did not disseminate adequate information to users of RHF and ORT respectively [32, 59]. However, three studies found that when health workers were trained on ORT management and knowledge, this was a facilitator to successful implementation [31, 47, 48]. Indeed, research in Mexico found that “the prescription of ORT for children younger than 5 years old increased from 31.4% to 59.4%” [47] (p442) following training of health workers [47]. The creation of ORT education programs for mothers/caregivers were also found to be a facilitator to implementation in four studies [49–51, 60]. A study in India assessing two phases of ORS promotion (phase I) and discontinuation (phase II) found that “following discontinuation of ORS packets, there was no decline in the use of ORT when mothers were educated to prepare a substitute solution with the household ingredients. Education on diarrhoeal management during the initial phase is likely to have improved the motivation among mothers to use ORT during sugar salt period” [49] (p200), emphasizing this review’s findings on the role of maternal/caregiver education programs for implementation. Health clinics providing education were also found to be a valuable asset to distributing information about ORS, thus acting as a facilitator to increase uptake [45]. Finally, training community members on preparation of ORT with ingredients available at home, also known as homemade ORT training, was cited as a facilitator to improving uptake as well [52].

#### Partnership engagement

*Engaging (process)*. Engaging refers to the use of social marketing, education, and similar strategies to draw appropriate individuals into the implementation and use of ORT. The dissemination of information and awareness pertaining to ORT through such methods was cited as a facilitator by 9 studies [23, 56, 61–67]. Of these 9 studies, 7 mentioned mass media campaigns as a facilitator to implementation, citing them as an effective means of increasing both knowledge and uptake of ORT in communities, especially when adapted to the local context [61–65, 67], or targeting a focal district [66]. Campaigns were noted as being more successful in increasing knowledge rather than practice, yet were commended as successful to implementation nonetheless. Additionally, a study conducted in Bangladesh noted that “mass awareness raising effort helped substantially shift community norms from restricted feeding to feeding to the use of home-made solution for dehydration correction” [23] (p5), further increasing RHF uptake through the use of mass-media campaigns.

*Cosmopolitanism (outer setting)*. This CFIR construct addresses the level of networking between and within various groups, as well as the engagement of and active participation with said groups. With respect to this construct, 2 of the 55 studies suggested that engaging both the private and public sector was a crucial facilitator to increasing uptake of ORS [68, 69]. Appointing private and public sectors as indicated in India and Ghana to oversee administration of ORS ensued various benefits, including increased access to ORS and zinc [68, 69]. Recent research suggested the advantage of supporting an even larger network, citing the essential role of non-government organizations (NGOs) along with private and public sector stewardship in increasing uptake of ORS [23].

*External policies and incentives (outer setting)*. Government funding and stewardship was noted as a facilitator in 4 of the 55 studies for the implementation of ORS, with these studies focusing on populations from India, Egypt, Uganda, Kenya and Nigeria acknowledging that programs funded by the government increased ORS uptake [30, 70–72]. Of these 4 studies, 3 mentioned that programs with enough funds to widely distribute ORS and train healthcare workers and pharmacists significantly increased utilization of ORS, whilst also increasing the accessibility and availability of the product [30, 70, 71]. Consequently, the government-led programs reduced unnecessary drug treatment [70] and increased utilization of public health services [30]. National and statewide ORS coverage programs to increase uptake of ORS was also described as a facilitator [72].

#### Design and acceptability

*Design*, *quality and packaging (intervention characteristics)*. Our research uncovered studies citing both negative and positive factors relating to the design and packaging of ORS [40, 41, 45, 71, 73] and its method of packaging [41, 71, 73], respectively. Concerning the design of the product, studies in Papua New Guinea and Nicaragua cited taste as a barrier [40, 45]. These 2 studies mentioned children’s aversion to ORS; its unpleasant taste negatively impacted its uptake [40, 45]. In contrast, 3 studies cited the method of packaging of the product as a facilitator to its implementation of ORS [41, 71, 73]. When ORS is packaged either separately or co-packaged with zinc in pouches, all containing instructional messages for the intended user, it ensues various benefits to the user, and is therefore a facilitator. Benefits of this packaging method include higher dissemination of information, better adherence to the treatment course, and increases in product uptake [41, 71, 73], which are also associated with lower utilization of unnecessary drugs and fluids [73].

*Other personal attributes (characteristics of individuals)*. Females were found to not be given ORS as frequently as their male counterparts in India, highlighting the sex divide where larger numbers of deaths among female children than male children were observed [74]. Low literacy of mothers in Haiti was also cited as a barrier, as it correlated with both uptake and low knowledge of ORT [27]. These authors indicated “The fact that literacy correlates with knowledge and early initiation of ORT, independent of place of residence, underscores the well-established finding that the better educated are more receptive to innovations” [27] (p94), concluding that populations with higher literacy rates are more amenable to the implementation of ORT [27].

*Adaptability (intervention characteristics)*. The ability to adapt ORT to different settings and cultures greatly affected implementation as a facilitator. Research in Ethiopia found that the adaptability of home-made RHF was accepted as a treatment because it was similar to a locally made cereal that was culturally familiar [75], stating that “compliance after 48 hours of treatment was found to be considerably better among caretakers providing the HC-ORT. This may in part be because of its similarity to a locally prepared cereal, known as buluka (Oromo) or atemeet (Amhara), thus enhancing its cultural acceptability” [75] (p1291-1292). This demonstrates how caretakers are more compliant to the treatment if it is made with familiar and culturally accepted ingredients such as cereal, which in turn improves uptake. Similarly, research in Bangladesh found that rice-based RHF was more culturally acceptable as this was similar to traditional foods made in the households [76]. This construct was also concerned with adapting essential elements or components related to ORT to tailor it to fit contextual and local needs of target populations. Of these elements, ORT’s workforce plays an important role in implementation. Implementing a village-based delivery system was very successful in increasing uptake in a study set in India [36]. This system utilized local volunteers and Village Headmen to deliver the product and disseminate ORT knowledge to the community [36]. Additionally, employing local workers was cited as a facilitator in India and Brazil [36, 77]. This employment system was found to be beneficial as it not only conferred higher acceptance and participation by local people, but was also a more feasible and economically advantageous means of implementation [36, 77].

## Discussion

This systematic review identified multiple barriers and facilitators to implementing ORT in LMICs across 55 articles published between 1981 to 2020. The identified barriers and facilitators are consistent with the types of barriers that have been described previously in other reviews during the past decade [10, 11, 14]. Our analysis was based on twenty-three countries with India, Bangladesh, Egypt, Nigeria, and South Africa having the most representation in available studies.

The availability of ORT supply is still a critical factor in ORT implementation, particularly related to ORS. Both facilitators and barriers were associated with availability of ORS, which is more problematic in rural areas than urban areas, pointing to earlier studies that have demonstrated differences in ORS supply and uptake between urban and rural populations [14, 78]. Factors affecting availability of ORS in rural and isolated areas do vary, and can include issues with supply, procurement and distribution [79]. These contributing factors are also not static and often times sporadic, thus highlighting that for ongoing interventions, accurate forecasted demand data can assist implementors to ensure sufficient coverage within areas of high demand and prevent overstocking within low-demand areas. The mix of strategies chosen must depend on local assets, opportunities, and resources that exist or could be mobilized. In situations where ORS is available, cost can be a barrier to purchase, thus limiting accessibility particularly among the poor and those living in rural areas [80]. Pricing of ORS product can affect ORS use, therefore ORS availability does not equal accessibility if users cannot access it due to high cost [14]. Conversely, too low of a price could disincentivize manufacturers and distributors and affect availability [81]. Interestingly, the unit price for ORS may be becoming less of a barrier for those who need it as this was less cited as a barrier in recent years (2000–2019), probably because of greater subsidization and investment in ORS supply, and the emergence of several facilitators including: implementing social franchising programs, an engaged private/public sector, and focused district interventions. However, due to the myriad of factors associated availability and accessibility that differ by local context, a better understanding of these factors by implementers and practitioners is critical for effective implementation.

Additionally, this study has determined that knowledge is still a factor in ORT uptake. Educating populations on the causes of childhood diarrhea and the importance of ORT are key means to establish and increase uptake. However, significant impacts on uptake rates cannot be made if challenges lie further upstream with healthcare workers that lack knowledge of diarrhea mortality and treatment or caregivers not familiar with it. For example, healthcare workers lacking knowledge of diarrhea mortality and treatment plans could be due to ineffective implementation of the Integrated Management of Childhood Illnesses (IMCI) strategy at health facilities, or ineffective community case management. In such contexts, use of ORT may become inadequate as the role that ORS plays in preventing child deaths caused by diarrhea or the methods of ORT administration are poorly supported. There have been calls for better understanding of family care seeking behavior to improve diarrhea case management in low- and-middle-income countries [82]. Specifically, barriers related to knowledge which were prominent in the 1980s and 90s (discouragement from health providers, unclear definition of a diarrheal episode, preference for alternative treatments, and poor education/training of workers), have featured less in recently published articles. This could imply that the increase in efforts to improve knowledge around efficacy of ORS to treat diarrhea have been successful. This would not be surprising as we have also seen an increase in the number of facilitators related to increasing knowledge around ORS particularly through training of community health care workers and mass media campaigns [10]. Furthermore, countries such as Sierra Leone have focused community mobilization to promote access to and awareness of ORT have also seen increases in ORT coverage [14]. This is a positive sign, however, due to the variability between LMIC’s socio-political and economic contexts, more still could be done. It will also be critical to assess the knowledge gaps in each local context to understand and tackle the specific root causes related to knowledge. The need to understand local settings and address any knowledge-related gaps to foster ORT uptake is emphasized in other studies similar to our own [4, 9].

Improving partnership engagement, including external policies and incentives, can lead to successful implementation of ORT in various settings. Although most countries in LMICs have some form of national policy on management of childhood diarrhea (e.g., 100% of countries in the South-East Asia Region, 93% in the African region [83]), there are sizable gaps between policy and implementation. IMCI strategies emphasize improving healthcare seeking behavior [84] and are implemented in LMICs; however, diarrhea case management indicators have not improved in tandem. For example, in a survey of 50 countdown priority countries looking at community case management (CCM), although 81% of these countries had CCM policies in place, ORS plus zinc, and full osmolarity ORS were distributed by CHWs in only 34% and 66% of these countries respectively [85]. In countries that might have policies related to diarrhea treatment, there could be policy implementation gaps. Highlighting policy alone is not sufficient but other factors related to engagement and government effectiveness may play a role in ORS uptake as well [86]. Scaling of ORS has been more successful in countries that have both national buy-in and collaborations between government and private sections [11]. Engagements of private providers, involvement of the non-government organizations and the private sector along with public sector stewardship can be instrumental in improving ORS uptake. This also includes government subsidized programs and combined NGO/private sector/public sector stewardship. Engaging both the private and public sectors is essential to ensure increased access to ORS and can alleviate the barriers resulting from supply and availability.

Design and acceptability of ORS and RHF are still critical factors in their uptake and implementation. Local communities and stakeholders must be part of the implementation strategies as community driven initiatives and local leaders are the ones to drive the process and buy into it. These stakeholders have a better understanding of the varied nuanced challenges and cultural barriers within local geographies and are equipped to provide advice and support implementation on designs to overcome these constraints. This is also significant for RHF and how they are communicated, branded, and prepared. We found that packaging matters. Pre-packaging ORT/zinc for mothers made them 1.82 times more likely to use ORS to treat their child’s diarrhea episode compared to mothers who were not exposed to the same kind of package [10]. Practitioners must therefore consider culturally adapted designs of ORS and RHF, explore barriers to acceptance amongst communities and take cultural norms into account. Working with community members is also important for successful implementation. Understanding the barriers and facilitators related to design and acceptability in local contexts can immediately inform ongoing program delivery and aid in future implementation plans, which is often one of the deciding factors on whether a strategy is successful or not.

The overall picture emerging from this analysis does not support claims that any major gains are to be made by completely re-designing ongoing projects with some “game-changing” shift in focus, pathways or outcomes across all countries. Rather, it points to an abundance of options for improvement, both through adjustments of ongoing program delivery and rebalancing of priorities in planned programs. Indeed, at least in theory from these findings, it is possible that in any given local context, a well-targeted, small series of process and delivery changes could presage cumulatively large and positive changes in performance indicators and selected outcomes. We suggest that implementation practitioners consider ways to use an implementation framework to conduct a self-assessment of their approaches considering barriers or facilitators that may be available. Our results support retaining a “holistic” approach which considers all options, and always remaining flexible to adapt as well as open to modifying, any specific program mix depending on the local context.

### Strengths and limitations

Throughout the screening process, 21 out of 1591 studies could not be screened for inclusion as their full-text articles were not available online. Although libraries that hold these articles were contacted, the COVID-19 pandemic created restrictions on providing interlibrary loans on print materials as well as access issues related to scanning services. A potential limitation is that these articles may contain relevant or important information.

In addition, our screening strategy did not pick up articles that matched the subject terms or keywords for the concepts of ORT, diarrhea, and children, but did not match the search terms we developed for the concept of implementation science. There are articles where titles/abstracts/subject terms did not explicitly mention “*implementation*”, “*evaluation*”, or “*adoption*”, or related terms like “*scale up*” or “*enabling environment*”. As a result, we missed several articles that should have been added to the list of eligible articles. However, we used this approach because our inclusion criteria specified that we wanted to retrieve implementation studies rather than evaluation studies, and we wanted articles written from the perspective of implementation science (that focused on the process and barriers and facilitators of implementation). So, for this reason, we included search terms targeting articles from this area, rather than searching for all articles related to ORT for the treatment of childhood diarrhea. In order to identify additional literature, a future systematic review could incorporate more information sources into the search strategy, including consultation with stakeholders and citation searches.

The inclusion of lower quality studies may have also lowered the confidence in some of our estimates. Of the studies scored by the MMAT, two were accorded a score of 2 or below. Nonetheless, inclusion of these studies allows for the capture of broader data. We found several examples of grey literature (i.e conference briefings) on ORT during the entire screening process in Covidence. These sources of information could have contained useful information for this review but were excluded due to the eligibility criteria (which specifically excluded grey literature) as outlined in our systematic review protocol (A PRISMA Checklist is included in S4 Appendix).

Finally, the selection of a determinant framework (CFIR) is a potential limitation as such frameworks have been criticized for their inadequacy in addressing causal mechanisms or how change takes place [87]. However, the use of the CFIR allowed the findings to be placed in the context of the wider implementation research literature [88, 89]. The CFIR assisted in triangulating information and also allowed us to conceptualize the barriers and facilitators in a more organized manner and the application of 10 out of 39 CFIR constructs.

## Conclusion

The results from this systematic review can inform the planning and design of new or ongoing evaluations, and the development of implementation strategies specifically aimed at overcoming common barriers and leveraging known facilitators. These key areas are (1) availability and accessibility, (2) knowledge, (3) partnership engagement, and (4) design and acceptability. The barriers and facilitators identified under the CFIR domains can be used to build knowledge on how to adapt ORT to national and local settings and to contribute to a better understanding of the implementation and use of ORT in LMICs. We note that barriers and facilitators were identified in all of the CFIR domains examined. This means that no simple, “silver bullet” approach to implementation aimed at either removing barriers or strengthening facilitators is recommended. Rather, practitioners and policy makers should invest in a mix of efforts to mitigate barriers but also promote enablers depending on the local implementation context. The prospects for scaling and sustaining ORT (after years of low use) will increase if implementation research informs local applications, implementers engage appropriate stakeholders and test assumptions around localized theories of change from interventions to expected outcomes.

## Supporting information

S1 File Search strings. (DOCX)

S2 File PRISMA checklist. (DOCX)

S1 Table Mixed-methods appraisal tool. (DOCX)

S2 Table Study characteristics. (DOCX)

S3 Table Barriers to ORT implementation. (DOCX)

S4 Table Facilitators to ORT implementation. (DOCX)
