# Supplementary material for: Barriers and facilitators to implementation of oral rehydration therapy in low- and middle-income countries: A systematic review
Source: PLoS One. 2021 Apr 22;16(4):e0249638. doi: 10.1371/journal.pone.0249638 (PMC8062013; doi:10.1371/journal.pone.0249638)
Supplement: S1 File — (DOCX) [file pone.0249638.s001.docx]

# **S1 File. Search Strings**

*Ovid MEDLINE Search Strategy*

|  | | | |
| --- | --- | --- | --- |
| # | Search Statement | Results | Annotation |
| 1 | exp Fluid Therapy/ | 19858 |  |
| 2 | fluid therap*.mp. | 21537 |  |
| 3 | oral rehydration*.mp. | 3030 |  |
| 4 | glucose-salt solution.mp. | 9 |  |
| 5 | exp Diarrhea/ | 52050 |  |
| 6 | diarrhea.mp. | 100640 |  |
| 7 | diarrhoea.mp. | 27061 |  |
| 8 | exp Child, Preschool/ | 902847 |  |
| 9 | exp Infant/ | 1122398 |  |
| 10 | child*.mp. | 2391648 |  |
| 11 | infant*.mp. | 1245875 |  |
| 12 | newborn*.mp. | 749337 |  |
| 13 | (under five adj5 (year* or age)).mp. | 2029 |  |
| 14 | (under-five adj5 (year* or age)).mp. | 2029 |  |
| 15 | exp Implementation Science/ | 287 |  |
| 16 | implement*.mp. | 470763 |  |
| 17 | adopt*.mp. | 248060 |  |
| 18 | evaluat*.mp. | 3747200 |  |
| 19 | scale-up.mp. | 11002 |  |
| 20 | scale up.mp. | 11002 |  |
| 21 | enabling environment*.mp. | 487 |  |
| 22 | 1 or 2 or 3 or 4 | 22954 |  |
| 23 | 5 or 6 or 7 | 115597 |  |
| 24 | 8 or 9 or 10 or 11 or 12 or 13 or 14 | 3045954 |  |
| 25 | 15 or 16 or 17 or 18 or 19 or 20 or 21 | 4276369 |  |
| 26 | 22 and 23 and 24 and 25 | 684 |  |
| 27 | limit 26 to (english language and humans and yr="1960 - 2020" and ("newborn infant (birth to 1 month)" or "infant (1 to 23 months)" or "preschool child (2 to 5 years)") and journal article) | 410 |  |

*Ovid EMBASE Search Strategy*

|  |  |  |  |
| --- | --- | --- | --- |
| # | Search Statement | Results | Annotation |
| 1 | exp oral rehydration therapy/ | 2637 |  |
| 2 | oral rehydration*.mp. | 6087 |  |
| 3 | fluid therap*.mp. | 23380 |  |
| 4 | glucose-salt solution.mp. | 12 |  |
| 5 | exp diarrhea/ | 261803 |  |
| 6 | diarrhea.mp. | 283693 |  |
| 7 | diarrhoea.mp. | 42738 |  |
| 8 | exp child/ | 2963686 |  |
| 9 | exp infant/ | 1140952 |  |
| 10 | child*.mp. | 2929671 |  |
| 11 | infant*.mp. | 992397 |  |
| 12 | newborn*.mp. | 738262 |  |
| 13 | (under five adj5 (year* or age)).mp. | 2760 |  |
| 14 | (under-five adj5 (year* or age)).mp. | 2760 |  |
| 15 | exp implementation science/ | 837 |  |
| 16 | implement*.mp. | 621979 |  |
| 17 | adopt*.mp. | 318445 |  |
| 18 | evaluat*.mp. | 5093923 |  |
| 19 | scale-up.mp. | 19697 |  |
| 20 | scale up.mp. | 19697 |  |
| 21 | enabling environment*.mp. | 595 |  |
| 22 | 1 or 2 or 3 or 4 | 28351 |  |
| 23 | 5 or 6 or 7 | 299181 |  |
| 24 | 8 or 9 or 10 or 11 or 12 or 13 or 14 | 3721692 |  |
| 25 | 15 or 16 or 17 or 18 or 19 or 20 or 21 | 5778485 |  |
| 26 | 22 and 23 and 24 and 25 | 768 |  |
| 27 | limit 26 to (human and english language and yr="1960 - 2020" and article and (infant <to one year> or child <unspecified age> or preschool child <1 to 6 years>)) | 345 |  |

*Ovid HealthSTAR Search Strategy*

| # | Search Statement | Results | Annotation |
| --- | --- | --- | --- |
| 1 | exp Fluid Therapy/ | 16355 |  |
| 2 | fluid therap*.mp. | 17060 |  |
| 3 | oral rehydration*.mp. | 2697 |  |
| 4 | glucose-salt solution.mp. | 8 |  |
| 5 | 1 or 2 or 3 or 4 | 18211 |  |
| 6 | exp Implementation Science/ | 243 |  |
| 7 | implement*.mp. | 351678 |  |
| 8 | adopt*.mp. | 148214 |  |
| 9 | evaluat*.mp. | 2511951 |  |
| 10 | scale-up.mp. | 6930 |  |
| 11 | scale up.mp. | 6930 |  |
| 12 | enabling environment*.mp. | 391 |  |
| 13 | exp Diarrhea/ | 34329 |  |
| 14 | diarrhea.mp. | 60723 |  |
| 15 | diarrhoea.mp. | 17891 |  |
| 16 | 13 or 14 or 15 | 70444 |  |
| 17 | exp Child, Preschool/ | 745954 |  |
| 18 | exp Infant/ | 893891 |  |
| 19 | exp Infant, Newborn/ | 493009 |  |
| 20 | child*.mp. | 1840696 |  |
| 21 | infant*.mp. | 949848 |  |
| 22 | newborn*.mp. | 509732 |  |
| 23 | (under five adj5 (year* or age)).mp. | 1807 |  |
| 24 | (under-five adj5 (year* or age)).mp. | 1807 |  |
| 25 | 17 or 18 or 19 or 20 or 21 or 22 or 23 or 24 | 2199294 |  |
| 26 | 6 or 7 or 8 or 9 or 10 or 11 or 12 | 2860856 |  |
| 27 | 5 and 16 and 25 and 26 | 653 |  |
| 28 | limit 27 to (humans and english language and ("newborn infant (birth to 1 month)" or "infant (1 to 23 months)" or "preschool child (2 to 5 years)") and yr="1960 - 2020" and journal article) | 426 |  |

*WEB OF SCIENCE Search Strategy*

TOPIC: ("fluid therap*" OR "oral rehydration*" OR "glucose-salt solution") *AND* TOPIC: (implement* OR adopt* OR evaluat* OR "scale up" OR "scale-up" OR "enabling environment*") *AND* TOPIC: (child* OR infant* OR newborn* OR "under-five" NEAR/5 year OR "under-five" NEAR/5 age OR "under five" NEAR/5 year OR "under five" NEAR/5 age) *AND* TOPIC: (diarrhea OR diarrhoea)

Refined by: DOCUMENT TYPES: ( ARTICLE ) AND LANGUAGES: ( ENGLISH )

Timespan: 1960-2020. Indexes: SCI-EXPANDED, SSCI, A&HCI, CPCI-S, CPCI-SSH, BKCI-S, BKCI-SSH, ESCI.

Number of Results: 304

*Scopus Search Strategy*

## ( TITLE-ABS-KEY ( *"fluid therap*"* OR *"oral rehydration*"* OR *"glucose-salt solution"* ) AND TITLE-ABS-KEY ( *implement** OR *adopt** OR *evaluat** OR *"scale up"* OR *scale-up* OR *"enabling environment*"* ) AND TITLE-ABS-KEY ( ( *child** OR *infant** OR *newborn** OR *"under five"* OR *under-five* ) W/5 ( *year* OR *age* ) ) AND TITLE-ABS-KEY ( *diarrhea* OR *diarrhoea* ) ) AND DOCTYPE ( *ar* ) AND PUBYEAR > *1959* AND PUBYEAR < *2021* AND ( LIMIT-TO ( DOCTYPE , *"ar"* ) ) AND ( LIMIT-TO ( LANGUAGE , *"English"* ) ) AND ( LIMIT-TO ( SRCTYPE , *"j"* ) )

Number of Results: 223
