# Supplementary material for: Barriers and facilitators to implementation of oral rehydration therapy in low- and middle-income countries: A systematic review
Source: PLoS One. 2021 Apr 22;16(4):e0249638. doi: 10.1371/journal.pone.0249638 (PMC8062013; doi:10.1371/journal.pone.0249638)
Supplement: S1 Table — (DOCX) [file pone.0249638.s003.docx]

**S1 Table. Mixed-Methods Appraisal Tool**

| Screening Questions | | | | | | | | | |  |
| --- | --- | --- | --- | --- | --- | --- | --- | --- | --- | --- |
| S1. Are there clear research questions? | | | | | | S2. Do the collected data allow to address the research questions? | | | |  |
| 1. Qualitative Studies | | | | | | | | | |  |
| 1.1. Is the qualitative approach appropriate to answer the research question? | 1.2. Are the qualitative data collection methods adequate to address the research question? | | 1.3. Are the findings adequately derived from the data? | | | 1.4. Is the interpretation of results sufficiently substantiated by data? | | 1.5. Is there coherence between qualitative data sources, collection, analysis and interpretation? | |  |
| 2. Randomized Control Trials | | | | | | | | | |  |
| 2.1. Is randomization appropriately performed? | 2.2. Are the groups comparable at baseline? | | 2.3. Are there complete outcome data? | | | 2.4. Are outcome assessors blinded to the intervention provided? | | 2.5 Did the participants adhere to the assigned intervention? | |  |
| 3. Non-Randomized Studies | | | | | | | | | |  |
| 3.1. Are the participants representative of the target population? | 3.2. Are measurements appropriate regarding both the outcome and intervention (or exposure)? | | 3.3. Are there complete outcome data? | | | 3.4. Are the confounders accounted for in the design and analysis? | | 3.5. During the study period, is the intervention administered (or exposure occurred) as intended? | |  |
| 4. Quantitative Descriptive Studies | | | | | | | | | |  |
| 4.1. Is the sampling strategy relevant to address the research question? | 4.2. Is the sample representative of the target population? | | 4.3. Are the measurements appropriate? | | | 4.4. Is the risk of nonresponse bias low? | | 4.5. Is the statistical analysis appropriate to answer the research question? | |  |
| 5. Mixed-Method Studies | | | | | | | | | |  |
| 5.1. Is there an adequate rationale for using a mixed methods design to address the research question? | 5.2. Are the different components of the study effectively integrated to answer the research question? | | 5.3. Are the outputs of the integration of qualitative and quantitative components adequately interpreted? | | | 5.4. Are divergences and inconsistencies between quantitative and qualitative results adequately addressed? | | 5.5. Do the different components of the study adhere to the quality criteria of each tradition of the methods involved? | |  |
| Author/Year | | Screening Questions | | | Type of Study | | Methodological Quality Criteria | | | |
| Akpede et al., 1997 [33] | | S1. Are there clear research questions? | | Yes | Qualitative | | 1.1 | | Yes | |
|  |  |  |  |  |  |  | 1.2 | | Yes | |
|  |  | S2. Do the collected data allow to address the research questions? | | Yes |  |  | 1.3 | | Yes | |
|  |  |  |  |  |  |  | 1.4 | | Yes | |
|  |  |  |  |  |  |  | 1.5 | | Yes | |
| Ali et al., 2017 [26] | | S1. | | Yes | Mixed Methods Studies | | 5.1 | | Yes | |
|  |  |  |  |  |  |  | 5.2 | | Yes | |
|  |  | S2. | | Yes |  |  | 5.3 | | Yes | |
|  |  |  |  |  |  |  | 5.4 | | Yes | |
|  |  |  |  |  |  |  | 5.5 | | Yes | |
| Aung et al., 2014 [31] | | S.1 | | Yes | Randomized Control Trial | | 2.1 | | Yes | |
|  |  |  |  |  |  |  | 2.2 | | Yes | |
|  |  | S.2 | | Yes |  |  | 2.3 | | Yes | |
|  |  |  |  |  |  |  | 2.4 | | No | |
|  |  |  |  |  |  |  | 2.5 | | Yes | |
| Baltazar et al., 2002 [29] | | S.1 | | Yes | Quantitative Descriptive Studies | | 4.1 | | Yes | |
|  |  |  |  |  |  |  | 4.2 | | Yes | |
|  |  |  |  |  |  |  | 4.3 | | Yes | |
|  |  | S.2 | | Yes |  |  | 4.4 | | Can’t Tell | |
|  |  |  |  |  |  |  | 4.5 | | Yes | |
|  |  |  |  |  |  |  | 4.5 | | Yes | |
| Bhan et al., 1988 [49] | | S.1 | | No | Qualitative Studies | | 1.1 | | Yes | |
|  |  |  |  |  |  |  | 1.2 | | Yes | |
|  |  | S.2 | | Yes |  |  | 1.3 | | Yes | |
|  |  |  |  |  |  |  | 1.4 | | Yes | |
|  |  |  |  |  |  |  | 1.5 | | Yes | |
| Bhandari et al., 2008 [68] | | S.1 | | Yes | Randomized Control Trial | | 2.1 | | Yes | |
|  |  |  |  |  |  |  | 2.2 | | Yes | |
|  |  |  |  |  |  |  | 2.3 | | Yes | |
|  |  |  |  |  |  |  | 2.4 | | Can’t Tell | |
|  |  | S.2 | | Yes |  |  | 2.5 | | Yes | |
| Billah et al., 2019 [23] | | S.1 | | Yes | Qualitative Studies | | 1.1 | | Yes | |
|  |  |  |  |  |  |  | 1.2 | | Yes | |
|  |  |  |  |  |  |  | 1.3 | | Yes | |
|  |  |  |  |  |  |  | 1.4 | | Yes | |
|  |  | S.2 | | Yes |  |  | 1.5 | | Yes | |
| Charyeva et al., 2015 [24] | | S.1 | | Yes | Quantitative Studies | | 4.1 | | Yes | |
|  |  |  |  |  |  |  | 4.2 | | Yes | |
|  |  | S.2 | | Yes |  |  | 4.3 | | Yes | |
|  |  |  |  |  |  |  | 4.4 | | Yes | |
|  |  |  |  |  |  |  | 4.5 | | Yes | |
| Chowdhury et al., 1997 [54] | | S.1 | | Yes | Qualitative | | 1.1 | | Yes | |
|  |  |  |  |  |  |  | 1.2 | | Yes | |
|  |  | S.2 | | Yes |  |  | 1.3 | | Yes | |
|  |  |  |  |  |  |  | 1.4 | | Yes | |
|  |  |  |  |  |  |  | 1.5 | | Yes | |
| Chowdhury et al., 1988 [46] | | S.1 | | Yes | Qualitative | | 1.1 | | Yes | |
|  |  |  |  |  |  |  | 1.2 | | Yes | |
|  |  | S.2 | | Yes |  |  | 1.3 | | Yes | |
|  |  |  |  |  |  |  | 1.4 | | Yes | |
|  |  |  |  |  |  |  | 1.5 | | Yes | |
| Chowdhury et al., 1988 [39] | | S.1 | | Yes | Qualitative | | 1.1 | | Yes | |
|  |  |  |  |  |  |  | 1.2 | | Yes | |
|  |  | S.2 | | Yes |  |  | 1.3 | | Yes | |
|  |  |  |  |  |  |  | 1.4 | | Yes | |
|  |  |  |  |  |  |  | 1.5 | | Yes | |
| Clow, 1985 [60] | | S.1 | | Yes | Quantitative Descriptive Study | | 4.1 | | Yes | |
|  |  |  |  |  |  |  | 4.2 | | Yes | |
|  |  | S.2 | | Yes |  |  | 4.3 | | Yes | |
|  |  |  |  |  |  |  | 4.4 | | Yes | |
|  |  |  |  |  |  |  | 4.5 | | Yes | |
| Cooke et al., 2013 [61] | | S.1 | | Yes | Qualitative | | 1.1 | | Yes | |
|  |  |  |  |  |  |  | 1.2 | | Yes | |
|  |  | S.2 | | Yes |  |  | 1.3 | | Yes | |
|  |  |  |  |  |  |  | 1.4 | | Yes | |
|  |  |  |  |  |  |  | 1.5 | | Yes | |
| Coreil and Genece, 1988 [27] | | S.1 | | Yes | Qualitative Studies | | 1.1 | | Yes | |
|  |  |  |  |  |  |  | 1.2 | | Yes | |
|  |  | S.2 | | Yes |  |  | 1.3 | | Yes | |
|  |  |  |  |  |  |  | 1.4 | | Yes | |
|  |  |  |  |  |  |  | 1.5 | | Yes | |
| Deb et al., 1985 [36] | | S.1 | | Yes | Qualitative Studies | | 1.1 | | Yes | |
|  |  |  |  |  |  |  | 1.2 | | Yes | |
|  |  | S.2 | | Yes |  |  | 1.3 | | Yes | |
|  |  |  |  |  |  |  | 1.4 | | Yes | |
|  |  |  |  |  |  |  | 1.5 | | Yes | |
| Dippenaar et al., 2005 [38] | | S.1 | | Yes | Qualitative Studies | | 1.1 | | Yes | |
|  |  |  |  |  |  |  | 1.2 | | Yes | |
|  |  | S.2 | | Yes |  |  | 1.3 | | Yes | |
|  |  |  |  |  |  |  | 1.4 | | Yes | |
|  |  |  |  |  |  |  | 1.5 | | Yes | |
| El-Khoury et al., 2016 [69] | | S.1 | | Yes | Qualitative Studies | | 1.1 | | Yes | |
|  |  |  |  |  |  |  | 1.2 | | Yes | |
|  |  | S.2 | | Yes |  |  | 1.3 | | Yes | |
|  |  |  |  |  |  |  | 1.4 | | Yes | |
|  |  |  |  |  |  |  | 1.5 | | Yes | |
| El-Mougi et al., 1986 [50] | | S.1 | | Yes | Qualitative Studies | | 1.1 | | Yes | |
|  |  |  |  |  |  |  | 1.2 | | Yes | |
|  |  | S.2 | | Yes |  |  | 1.3 | | Yes | |
|  |  |  |  |  |  |  | 1.4 | | Yes | |
|  |  |  |  |  |  |  | 1.5 | | Yes | |
| el-Rafie et al., 1990 [70] | | S.1 | | Yes | Mixed Methods Studies | | 5.1 | | Yes | |
|  |  |  |  |  |  |  | 5.2 | | Yes | |
|  |  | S.2 | | Yes |  |  | 5.3 | | Yes | |
|  |  |  |  |  |  |  | 5.4 | | Yes | |
|  |  |  |  |  |  |  | 5.5 | | Yes | |
| Frankel and Lehmann, 1984 [40] | | S.1 | | Yes | Mixed Methods Studies | | 5.1 | | Can’t Tell | |
|  |  |  |  |  |  |  | 5.2 | | Yes | |
|  |  | S.2 | | Yes |  |  | 5.3 | | Yes | |
|  |  |  |  |  |  |  | 5.4 | | No | |
|  |  |  |  |  |  |  | 5.5 | | Can’t Tell | |
| Gebremedhin et al., 2016 [41] | | S.1 | | Yes | Randomized Control Trials | | 2.1 | | Yes | |
|  |  |  |  |  |  |  | 2.2 | | Yes | |
|  |  | S.2 | | Yes |  |  | 2.3 | | Yes | |
|  |  |  |  |  |  |  | 2.4 | | No | |
|  |  |  |  |  |  |  | 2.5 | | Yes | |
| Gibbons et al., 1994  [45] | | S.1 | | Yes | Mixed Methods Studies | | 5.1 | | Yes | |
|  |  | S.2 | | Yes |  |  | 5.2 | | Yes | |
|  |  |  |  |  |  |  | 5.3 | | Yes | |
|  |  |  |  |  |  |  | 5.4 | | Can’t Tell | |
|  |  |  |  |  |  |  | 5.5 | | Yes | |
| Greenough and Khin-Maung-U, 1991 [32] | | S.1 | | Yes | Quantitative Descriptive Studies | | 4.1 | | Yes | |
|  |  |  |  |  |  |  | 4.2 | | Yes | |
|  |  | S.2 | | Yes |  |  | 4.3 | | Yes | |
|  |  |  |  |  |  |  | 4.4 | | Yes | |
|  |  |  |  |  |  |  | 4.5 | | Yes | |
|  |  |  |  |  |  |  | 4.4 | | Yes | |
|  |  |  |  |  |  |  | 4.5 | | Yes | |
| Gutierrez et al., 1994 [47] | | S.1 | | Yes | Quantitative Descriptive Studies | | 4.1 | | Yes | |
|  |  |  |  |  |  |  | 4.2 | | Yes | |
|  |  | S.2 | | Yes |  |  | 4.3 | | Yes | |
|  |  |  |  |  |  |  | 4.4 | | Yes | |
|  |  |  |  |  |  |  | 4.5 | | Yes | |
| Gutierrez et al., 1996 [62] | | S.1 | | Yes | Quantitative Descriptive Studies | | 4.1 | | Yes | |
|  |  |  |  |  |  |  | 4.2 | | Yes | |
|  |  | S.2 | | Yes |  |  | 4.3 | | Yes | |
|  |  |  |  |  |  |  | 4.4 | | Yes | |
|  |  |  |  |  |  |  | 4.5 | | Yes | |
| Habib et al., 2013 [73] | | S.1 | | Yes | Randomized Control Trials | | 2.1 | | Yes | |
|  |  |  |  |  |  |  | 2.2 | | Yes | |
|  |  | S.2 | | Yes |  |  | 2.3 | | Yes | |
|  |  |  |  |  |  |  | 2.4 | | No | |
|  |  |  |  |  |  |  | 2.5 | | Yes | |
| Hall-Clifford and Amerson, 2017 [52] | | S.1 | | Yes | Qualitative Studies | | 1.1 | | Yes | |
|  |  |  |  |  |  |  | 1.2 | | Yes | |
|  |  | S.2 | | Yes |  |  | 1.3 | | Yes | |
|  |  |  |  |  |  |  | 1.4 | | Yes | |
|  |  |  |  |  |  |  | 1.5 | | Yes | |
| Heymann et al., 1990 [48] | | S.1 | | Yes | Quantitative Descriptive Studies | | 4.1 | | Yes | |
|  |  |  |  |  |  |  | 4.2 | | Yes | |
|  |  | S.2 | | Yes |  |  | 4.3 | | Yes | |
|  |  |  |  |  |  |  | 4.4 | | Yes | |
|  |  |  |  |  |  |  | 4.5 | | Yes | |
| Howteerakul et al., 2003 [42] | | S.1 | | Yes | Mixed Methods Studies | | 5.1 | | Yes | |
|  |  |  |  |  |  |  | 5.2 | | Yes | |
|  |  | S.2 | | Yes |  |  | 5.3 | | Yes | |
|  |  |  |  |  |  |  | 5.4 | | Yes | |
|  |  |  |  |  |  |  | 5.5 | | Yes | |
| Kassaye et al., 1994 [75] | | S.1 | | Yes | Randomized Control Trial | | 2.1 | | Yes | |
|  |  |  |  |  |  |  | 2.2 | | Yes | |
|  |  | S.2 | | Yes |  |  | 2.3 | | Yes | |
|  |  |  |  |  |  |  | 2.4 | | Yes | |
|  |  |  |  |  |  |  | 2.5 | | Yes | |
| Kassegne et al., 2011 [63] | | S.1 | | Yes | Quantitative Descriptive Studies | | 4.1 | | No | |
|  |  |  |  |  |  |  | 4.2 | | No | |
|  |  | S.2 | | Yes |  |  | 4.3 | | Yes | |
|  |  |  |  |  |  |  | 4.4 | | Yes | |
|  |  |  |  |  |  |  | 4.5 | | Yes | |
| Kenya et al., 1990 [64] | | S.1 | | Yes | Non-Randomized Control Trials | | 3.1 | | Yes | |
|  |  |  |  |  |  |  | 3.2 | | Yes | |
|  |  | S.2 | | Yes |  |  | 3.3 | | Yes | |
|  |  |  |  |  |  |  | 3.4 | | Yes | |
|  |  |  |  |  |  |  | 3.5 | | Yes | |
| Kielmann et al., 1985 [25] | | S.1 | | Yes | Non-Randomized Control Trials | | 3.1 | | Yes | |
|  |  |  |  |  |  |  | 3.2 | | Yes | |
|  |  | S.2 | | Yes |  |  | 3.3 | | Yes | |
|  |  |  |  |  |  |  | 3.4 | | Can’t Tell | |
|  |  |  |  |  |  |  | 3.5 | | Yes | |
| Kielmann et al., 1986 [57] | | S.1 | | Yes | Non-Randomized Control Trials | | 3.1 | | Yes | |
|  |  |  |  |  |  |  | 3.2 | | Yes | |
|  |  | S.2 | | Yes |  |  | 3.3 | | Yes | |
|  |  |  |  |  |  |  | 3.4 | | Can’t Tell | |
|  |  |  |  |  |  |  | 3.5 | | Yes | |
| Kumar et al., 2015 [30] | | S.1 | | Yes | Qualitative Studies | | 1.1 | | Yes | |
|  |  |  |  |  |  |  | 1.2 | | Yes | |
|  |  | S.2 | | Yes |  |  | 1.3 | | Yes | |
|  |  |  |  |  |  |  | 1.4 | | Yes | |
|  |  |  |  |  |  |  | 1.5 | | Yes | |
| Kumar et al., 1987 [74] | | S.1 | | Yes | Non-Randomized Control Trials | | 3.1 | | Yes | |
|  |  |  |  |  |  |  | 3.2 | | Yes | |
|  |  | S.2 | | Yes |  |  | 3.3 | | Yes | |
|  |  |  |  |  |  |  | 3.4 | | Can’t Tell | |
|  |  |  |  |  |  |  | 3.5 | | Yes | |
| Kumar et al., 1989 [55] | | S.1 | | Yes | Quantitative Descriptive Studies | | 4.1 | | Yes | |
|  |  |  |  |  |  |  | 4.2 | | Yes | |
|  |  | S.2 | | Yes |  |  | 4.3 | | Yes | |
|  |  |  |  |  |  |  | 4.4 | | Yes | |
|  |  |  |  |  |  |  | 4.5 | | Yes | |
| Lam et al., 2019 [66] | | S.1 | | Yes | Quantitative Descriptive Studies | | 4.1 | | Yes | |
|  |  |  |  |  |  |  | 4.2 | | Yes | |
|  |  | S.2 | | Yes |  |  | 4.3 | | Yes | |
|  |  |  |  |  |  |  | 4.4 | | Yes | |
|  |  |  |  |  |  |  | 4.5 | | Yes | |
| Lam et al., 2019 [71] | | S.1 | | Yes | Mixed Methods Studies | | 5.1 | | Yes | |
|  |  |  |  |  |  |  | 5.2 | | Yes | |
|  |  | S.2 | | Yes |  |  | 5.3 | | Yes | |
|  |  |  |  |  |  |  | 5.4 | | Yes | |
|  |  |  |  |  |  |  | 5.5 | | Yes | |
| Lam et al., 2019 [65] | | S.1 | | Yes | Quantitative Descriptive Studies | | 4.1 | | Yes | |
|  |  |  |  |  |  |  | 4.2 | | Yes | |
|  |  | S.2 | | Yes |  |  | 4.3 | | Yes | |
|  |  |  |  |  |  |  | 4.4 | | Yes | |
|  |  |  |  |  |  |  | 4.5 | | Yes | |
| Langsten and Hill, 1995 [56] | | S.1 | | Yes | Quantitative Descriptive Studies | | 4.1 | | Yes | |
|  |  |  |  |  |  |  | 4.2 | | Yes | |
|  |  | S.2 | | Yes |  |  | 4.3 | | Yes | |
|  |  |  |  |  |  |  | 4.4 | | Yes | |
|  |  |  |  |  |  |  | 4.5 | | Yes | |
| MacDonald et al., 2007 [37] | | S.1 | | Yes | Quantitative Descriptive Studies | | 4.1 | | Yes | |
|  |  |  |  |  |  |  | 4.2 | | Yes | |
|  |  | S.2 | | Yes |  |  | 4.3 | | Yes | |
|  |  |  |  |  |  |  | 4.4 | | Can’t Tell | |
|  |  |  |  |  |  |  | 4.5 | | Yes | |
| Maken et al., 2017 [43] | | S.1 | | Yes | Quantitative Descriptive Studies | | 4.1 | | Yes | |
|  |  |  |  |  |  |  | 4.2 | | Yes | |
|  |  | S.2 | | Yes |  |  | 4.3 | | Yes | |
|  |  |  |  |  |  |  | 4.4 | | Yes | |
|  |  |  |  |  |  |  | 4.5 | | Yes | |
| Mathur et al., 2019 [34] | | S.1 | | Yes | Quantitative Descriptive Studies | | 4.1 | | Yes | |
|  |  |  |  |  |  |  | 4.2 | | Yes | |
|  |  | S.2 | | Yes |  |  | 4.3 | | Yes | |
|  |  |  |  |  |  |  | 4.4 | | Yes | |
|  |  |  |  |  |  |  | 4.5 | | Yes | |
| Miller and Hirschhorn, 1995 [67] | | S.1 | | Yes | Quantitative Descriptive Studies | | 4.1 | | Yes | |
|  |  |  |  |  |  |  | 4.2 | | Yes | |
|  |  | S.2 | | Yes |  |  | 4.3 | | Yes | |
|  |  |  |  |  |  |  | 4.4 | | Yes | |
|  |  |  |  |  |  |  | 4.5 | | Yes | |
| Mull and Mull, 1988 [28] | | S.1 | | Yes | Qualitative Studies | | 1.1 | | Yes | |
|  |  |  |  |  |  |  | 1.2 | | Yes | |
|  |  | S.2 | | Yes |  |  | 1.3 | | Yes | |
|  |  |  |  |  |  |  | 1.4 | | Yes | |
|  |  |  |  |  |  |  | 1.5 | | Yes | |
| Nations et al., 1988 [77] | | S.1 | | Yes | Qualitative Studies | | 1.1 | | Yes | |
|  |  |  |  |  |  |  | 1.2 | | Yes | |
|  |  | S.2 | | Yes |  |  | 1.3 | | Yes | |
|  |  |  |  |  |  |  | 1.4 | | Yes | |
|  |  |  |  |  |  |  | 1.5 | | Yes | |
| Ogbo et al., 2014 [58] | | S.1 | | Yes | Mixed Methods Studies | | 5.1 | | Yes | |
|  |  |  |  |  |  |  | 5.2 | | Yes | |
|  |  | S.2 | | Yes |  |  | 5.3 | | Yes | |
|  |  |  |  |  |  |  | 5.4 | | Yes | |
|  |  |  |  |  |  |  | 5.5 | | Yes | |
| Okeke et al., 1996 [53] | | S.1 | | Yes | Qualitative Studies | | 1.1 | | Yes | |
|  |  |  |  |  |  |  | 1.2 | | Yes | |
|  |  | S.2 | | Yes |  |  | 1.3 | | Yes | |
|  |  |  |  |  |  |  | 1.4 | | Yes | |
|  |  |  |  |  |  |  | 1.5 | | Yes | |
| Raghu et al., 1995 [35] | | S.1 | | Yes | Quantitative Descriptive Studies | | 4.1 | | Yes | |
|  |  |  |  |  |  |  | 4.2 | | Yes | |
|  |  | S.2 | | Yes |  |  | 4.3 | | Yes | |
|  |  |  |  |  |  |  | 4.4 | | Yes | |
|  |  |  |  |  |  |  | 4.5 | | Yes | |
| Rahman et al., 1985 [76] | | S.1 | | Yes | Qualitative Studies | | 1.1 | | Yes | |
|  |  |  |  |  |  |  | 1.2 | | Yes | |
|  |  | S.2 | | Yes |  |  | 1.3 | | Yes | |
|  |  |  |  |  |  |  | 1.4 | | Yes | |
|  |  |  |  |  |  |  | 1.5 | | Yes | |
| Ronsmans et al., 1991 [51] | | S.1 | | Yes | Quantitative Descriptive Studies | | 4.1 | | Yes | |
|  |  |  |  |  |  |  | 4.2 | | Yes | |
|  |  | S.2 | | Yes |  |  | 4.3 | | Yes | |
|  |  |  |  |  |  |  | 4.4 | | Yes | |
|  |  |  |  |  |  |  | 4.5 | | Yes | |
| Schroder et al., 2019 [72] | | S.1 | | Yes | Quantitative Descriptive Studies | | 4.1 | | Yes | |
|  |  |  |  |  |  |  | 4.2 | | Yes | |
|  |  | S.2 | | Yes |  |  | 4.3 | | Yes | |
|  |  |  |  |  |  |  | 4.4 | | Yes | |
|  |  |  |  |  |  |  | 4.5 | | Yes | |
| Sircar et al., 1991 [59] | | S.1 | | Yes | Quantitative Descriptive Studies | | 4.1 | | Yes | |
|  |  |  |  |  |  |  | 4.2 | | Yes | |
|  |  | S.2 | | Yes |  |  | 4.3 | | Yes | |
|  |  |  |  |  |  |  | 4.4 | | Yes | |
|  |  |  |  |  |  |  | 4.5 | | Yes | |
| Touchette et al., 1994 [44] | | S.1 | | Yes | Qualitative Studies | | 1.1 | | Yes | |
|  |  |  |  |  |  |  | 1.2 | | Yes | |
|  |  | S.2 | | Yes |  |  | 1.3 | | Yes | |
|  |  |  |  |  |  |  | 1.4 | | Yes | |
|  |  |  |  |  |  |  | 1.5 | | Yes | |
