# Supplementary material for: Barriers and facilitators to implementation of oral rehydration therapy in low- and middle-income countries: A systematic review
Source: PLoS One. 2021 Apr 22;16(4):e0249638. doi: 10.1371/journal.pone.0249638 (PMC8062013; doi:10.1371/journal.pone.0249638)
Supplement: S2 Table — (DOCX) [file pone.0249638.s004.docx]

**S2 Table. Study Characteristics**

| Author/Year | Country | Intervention/Non-intervention | Methods | Participants (n) | Objective | MMAT Score |
| --- | --- | --- | --- | --- | --- | --- |
| Akpede et al., 1997  [33] | Nigeria | Non-intervention | Focus group discussions, Structured questionnaire-based survey | Sampled population from two ethnic groups, the Kanuris and the Buras. 260 respondents from the ethnic groups (130 from urban and 130 from rural areas). | To investigate awareness and knowledge of oral rehydration therapy and preparation abilities of salt-sugar solution. | 4 |
| Ali et al., 2017  [26] | India, Uttar Pradesh | Intervention | Survey, Focus group discussions, Interviews | Mothers/caregivers from four districts (n=1350 households). | To analyze the critical gaps at the public health system and community levels for the effective implementation of comprehensive diarrhea control. | 4 |
| Aung et al., 2014  [31] | Burma, Myanmar | Intervention | Household surveys | Sample population from 104 rural villages (n=1250 households). | To conduct a randomized controlled trial to increase ORS plus zinc uptake in rural Myanmar, via a social  franchising program. | 3 |
| Baltazar et al., 2002  [29] | Philippines | Intervention | National health statistic reports, Household surveys, Health facility surveys | General population. | To evaluate the impact of the National Control of Diarrhoeal Disease Programme. | 3 |
| Bhan et al., 1988  [49] | India | Intervention | Monitoring/observations by data collectors | Children (n=621). | To observe an oral rehydration therapy program introduced to a rural community in India for changes in treatment. | 4 |
| Bhandari et al., 2008  [68] | Haryana, India | Intervention | Interviews, Surveys, Cross-sectional surveys | 6 clusters of people from primary health centres, each with (n= 30,000). | To evaluate if education about zinc supplements and provision of zinc supplements to caregivers is effective in the treatment of acute diarrhea and affects use of oral rehydration salts. | 3 |
| Billah et al., 2019  [23] | Bangladesh | Non-intervention | Interviews, document reviews, Lives Saved Tool (LiST), Systematic search of literature | General Population. | To explore the drivers that helped Bangladesh achieve a large reduction in childhood deaths due to diarrheal disease in recent decades. | 4 |
| Charyeva et al., 2015  [24] | Northern Nigeria | Intervention | Surveys, Review of service statistics, Observations | ORT corner users (n = 110), ORT corner non-users (n = 119), ORT corner providers [n = 21], health facility providers (n = 23). | To introduce ORT corners to health facilities to provide treatment and equip caregivers with necessary skills in case management of diarrhea and diarrhea prevention. | 4 |
| Chowdhury et al., 1997  [54] | Bangladesh | Non-intervention | Interviews with questionnaire, Observation | Population from 90 villages [n=9000 households], village doctors [n=237], pharmacy attendants [n=296], pharmacy/ grocery shops [n=495], government outreach workers [n=306], government facilities [n=152]. | To assess knowledge of ORT preparation, its local availability and its use for the management of diarrhea. | 4 |
| Chowdhury et al., 1988  [46] | Bangladesh | Intervention | Household community survey, Interviews, Focus group discussions | Two villages [n=178 and n=422 for each], included users and non-users of lobon gur solution. | To evaluate a program where over a third of the households in Bangladesh were taught the preparation and use of an ORS made from lobon (common salt) and gur (unrefined sugar). | 4 |
| Chowdhury et al., 1988  [39] | Bangladesh | Intervention. | In-depth study of villages, Community survey, Interviews, Focus group discussions. | Households that received training [n=7000]. | To evaluate a program where mothers in over 5 million Bangladeshi households were taught how to prepare and use an oral rehydration solution containing lobon (local salt) and gur (unrefined sugar). | 4 |
| Clow, 1985  [60] | Kingdom of Tonga | Intervention | Data from statistics from ministry of health, Analysis of hospital statistics, Evaluation of program | General population [n=60000]. | To assess morbidity, mortality, and admission to hospital after implementation of an ORT training program. | 4 |
| Cooke et al., 2013  [61] | South Africa | Non-intervention | Interviews, Descriptive statistics | Children with diarrhea at a Tygerberg Children’s Hospital. | To document pre-hospital home and primary care management of diarrhea, and certain risk factors and complications of diarrhea. | 4 |
| Coreil and Genece, 1988  [27] | Haiti | Non-intervention | Interviews, Ethnographic study of villages | Mothers/caretakers [n=300], Mothers for interview [n=22], Families [n=1714]. | To report findings from a study of mothers’ knowledge and use of ORT for childhood diarrhea. | 4 |
| Deb et al., 1985  [36] | India | Intervention | Survey | Sample population from four villages [n=6410]. | To implement ORT in four villages through six locally recruited volunteers. | 4 |
| Dippenaar et al., 2005  [38] | South Africa | Non-intervention | Questionnaire | Caregivers [n=597]. | To evaluate caregivers’ knowledge of, attitudes to and use of homemade sugar and salt solution. | 4 |
| El-Khoury et al., 2016  [69] | Ghana | Intervention | Household surveys, Interviews | Caregivers [n=750]. | To evaluate a program that partnered with local pharmaceutical firms. | 4 |
| El-Mougi et al., 1986  [50] | Egypt | Intervention | Interviews | Mothers trained in program [n=100]. | To address the issue of assessing the effectiveness of the teaching mothers to change their knowledge and attitudes. | 4 |
| el-Rafie et al., 1990  [70] | Egypt | Intervention | Civic registration data, Service statistics, Household surveys | Sample population from urban and rural clusters. | To assess the effect of the National Control of Diarrheal Diseases Project on infant and childhood mortality. | 4 |
| Frankel and Lehmann, 1984  [40] | Papua New Guinea | Intervention | Monthly surveillance of population, Demographic surveillance | The Huli population [n=26000] in 1972, and [n=998] in 1978, and [n=2600] in 1979. | To evaluate the impact of an ORT program for diarrheal disease through continuing surveillance. | 1 |
| Gebremedhin et al., 2016  [41] | Ethiopia | Intervention | Statistical analysis | Children 6-59 months of age with diarrhea. | To evaluate whether co-packing using a plastic pouch can enhance the joint adherence to the treatment or not. | 3 |
| Gibbons et al., 1994  [45] | Nicaragua | Non-intervention | Health survey, Statistics, Interviews | Female caregivers [n=155 households]. | To ask caretakers about their knowledge, attitudes, and practices in treating diarrhea in children younger than age 5. | 4 |
| Greenough and Khin-Maung-U, 1991  [32] | Bangladesh | Intervention | Case follow-ups, Field trial study | Mothers of children less than 4 years  [n=305], and 7 villages [n=10,450]. | To analyze field studies about ORT training. | 4 |
| Gutierrez et al., 1994  [47] | Mexico City | Intervention | Interview, Group discussions, Evaluations | Two medical units with middle and lower class workers and their families [n=150,000], physicians [n=69]. | To design a strategy to decrease drug prescription and increase ORT use acute diarrhea treatment. | 4 |
| Gutierrez et al., 1996  [62] | Mexico | Non-intervention | Secondary data analysis | General Population. | To analyze mortality trends from diarrhoeal diseases among under-5-year-olds in Mexico between 1978 and 1993 in relation to the impact of education, basic sanitation, and selected medical care practices. | 4 |
| Habib et al., 2013  [73] | Pakistan | Intervention | Survey, Surveillance | Children under five years and their mothers [n=3000], Physicians, Traditional Healers, Quack Practitioners and health care providers. | To evaluate the acceptability, feasibility and impact of diarrhea pack on diarrhea burden. | 3 |
| Hall-Clifford and Amerson, 2017  [52] | Guatemala | Intervention | Interviews, Focus group discussions | A rural community [n=15000], and local stakeholders. | To co-design an ORT/ZS training program for community members with local health promoters. | 4 |
| Heymann et al., 1990  [48] | Malawi | Intervention | Paediatric inpatient records reviewed | Paediatric inpatients [n = 3495]. | To assess the outcomes of refresher training of paediatric staff in ORT and the establishment of an oral rehydration unit. | 4 |
| Howteerakul et al., 2003  [42] | Thailand | Non-intervention | Prospective clinical audit, Observations of treated cases, Interviews | Physicians. | To explore Thai physicians’ rationales about their prescribing practices for treating childhood diarrhoea. | 4 |
| Kassaye et al., 1994  [75] | Ethiopia | Intervention | Observations, Statistical analysis | Children under 5 years with acute childhood diarrhea [n = 291]. | To compare the effectiveness of prepackaged ORS vs homemade cereal based ORT in the treatment of diarrhea. | 4 |
| Kassegne et al., 2011  [63] | Burundi | Intervention | Household surveys | Females of reproductive age [15-49], caregivers. | To assess the use of a social marketing intervention on promoting the use of ORASEL. | 2 |
| Kenya et al., 1990  [64] | Kenya | Intervention | Survey, Statistical analysis | General population of Kakamega District [n = 1,030,887]. | To describe an ORT intervention campaign that used mass communication techniques to promote utilization of ORT. | 4 |
| Kielmann et al., 1985  [25] | Egypt | Intervention | Household surveys, Verbal Autopsy reports, Treatment and referral records | Children under the age of 5 [n = 28,850]. | To evaluate the effectiveness and replicability of a treatment regimen for the control of deaths from diarrhea in all districts covered by the "Strengthening Rural Health Delivery" project. | 3 |
| Kielmann et al., 1986  [57] | Egypt | Intervention | Surveys | Children under the age of 5 [n = 28,850]. | To identify ways to overcome ORT constraints related to logistics, supplies and community participations. | 3 |
| Kumar et al., 2015  [30] | India | Intervention | Program monitoring data, government surveys, secondary data sources | Children aged 2-59 months [n=5370014 in Gujarat, n=4338314 in Uttar Pradesh, n=13211546 in Bihar]. | To evaluate a project implemented to improve service delivery for childhood diarrhea management through the public health sector. | 4 |
| Kumar et al., 1987  [74] | India | Intervention | Surveillance | Children under 6 years of age from 69 villages. | To evaluate the effective reduction in diarrhea and utilization of WHO-recommended ORS in communities. | 3 |
| Kumar et al., 1989  [55] | India | Intervention | Structured observations | Sample population from 171 villages. | To evaluate the success of a government launched training program to educate doctors and health workers on treatment of patients with acute diarrhea. | 4 |
| Lam et al., 2019  [66] | Nigeria | Intervention | Household surveys | Children under five who had diarrhea in the past two weeks. | To evaluate a comprehensive program aimed at increasing treatment of diarrhea with ORS. | 4 |
| Lam et al., 2019  [71] | Uganda | Intervention | Surveys, Secondary analysis of government surveys and documents | General population. | To evaluate a program aimed at increasing treatment of diarrhea with ORS and zinc, by increasing their coverage. | 4 |
| Lam et al., 2019  [65] | India | Intervention | Household surveys | Caregivers. | To estimate the effect of program activities on ORS and combined ORS and zinc use. | 4 |
| Langsten and Hill, 1995  [56] | Egypt | Intervention | Household survey | Population from 12 rural villages. | To assess past successful oral rehydration programs. | 4 |
| MacDonald et al., 2007  [37] | Indonesia | Non-intervention | Interviews | Mothers [n=100]. | To assess whether mothers’ understanding of diarrhea-related dehydration influenced their use of ORS in home treatment. | 3 |
| Maken et al., 2017  [43] | Pakistan | Non-intervention | Questionnaire | Mothers whose children had diarrhea within the past 3 months [n=350]. | To address knowledge-practice gaps about management of diarrhea. | 4 |
| Mathur et al., 2019  [34] | India | Non-intervention | Interviews | Mothers and caregivers from nine villages and four slums. | To improve diarrheal case management using community participation and drug utilization research. | 4 |
| Miller and Hirschhorn, 1995  [67] | Egypt | Intervention | Surveys | General population. | To assess campaign focused on lowering mortality from diarrheal disease. | 4 |
| Mull and Mull, 1988  [28] | Pakistan | Non-intervention | Interviews, Consultations | Rural mothers [n=57]. | To analyze diarrhea-related traditional health beliefs and practices among rural and illiterate people to suggest important implications for ORT programs currently being launched. | 4 |
| Nations et al., 1988  [77] | Brazil | Intervention | Interviews | Mothers/caretakers. | To assess the role of healers in the promotion of ORT. | 4 |
| Ogbo et al., 2014  [58] | Nigeria | Non-intervention | Self-administered questionnaire, Simulated patient visits | Pharmacists [n=206]. | To evaluate knowledge and attitudes of community pharmacists in the management of diarrhea. | 4 |
| Okeke et al., 1996  [53] | Nigeria | Non-intervention | Interviews | Private medical practitioners [n=91]. | To assess the knowledge, attitude and practice of medical practitioners regarding ORT. | 4 |
| Raghu et al., 1995  [35] | India | Non-intervention | Interviews | Medical practitioners [n-48], pharmacists [n=56] and mothers of children with diarrhoea [n=55]. | To assess individual’s awareness of and attitudes towards ORT. | 4 |
| Rahman et al., 1985  [76] | Bangladesh | Non-intervention | Interviews, Field surveillance, Case follow-up, Rice-salt OR solution sample analysis | Mothers [n=305]. | To assess mothers’ ability to prepare and use rice-salt ORS. | 4 |
| Ronsmans et al., 1991  [51] | Bangladesh | Non-intervention | Demographic surveillance, Surveys | Community health practitioners, children with diarrhea. | To assess types of care provided for the management of dysentery. | 4 |
| Schroder et al., 2019  [72] | India, Kenya, Nigeria, Uganda | Intervention | Household surveys, Public facility audits, Private outlet surveys | General population. | To analyze the program results and lessons learned to inform other countries aiming to scale-up ORS and zinc. | 4 |
| Sircar et al., 1991  [59] | India | Intervention | Surveys | Children under 5 years and health workers. | To assess the impact of a 3-tier strategy for implementation of ORT. | 4 |
| Touchette et al., 1994  [44] | South Africa | Non-intervention | Observations | Mothers. | To measure the quantity of fluid unsupervised mothers gave their children at home. | 4 |
