# Supplementary material for: Barriers and facilitators to implementation of oral rehydration therapy in low- and middle-income countries: A systematic review
Source: PLoS One. 2021 Apr 22;16(4):e0249638. doi: 10.1371/journal.pone.0249638 (PMC8062013; doi:10.1371/journal.pone.0249638)
Supplement: S3 Table — (DOCX) [file pone.0249638.s005.docx]

**S3 Table. Barriers to ORT Implementation**

| **Barrier** | **Year** | **Geography** | **ORT/ORS/RHF** | **CFIR Framework** |
| --- | --- | --- | --- | --- |
| **Low knowledge of ORT** | | | | Knowledge and Beliefs about the Intervention (Characteristics of Individuals) |
| *“It is alarming that approximately 40 to 80% of the respondents in the survey either had no idea of ORT or have an inappropriate perception of the function of ORT. Lack of knowledge of its purpose can be impediment to ORT use.” [33]* | 1997 | Nigeria | RHF |  |
| *“In this study, the majority (64%) of mothers/caregivers (Figure 2b) perceived that syrups and tablets provided by healthcare providers are effective treatments for diarrhea. The knowledge of ORS and its benefits for childhood diarrhea was poor in our settings (28%, Figure 2b), which is in contrast to the findings of other studies conducted in India” [34]* | 2019 | India | ORS |  |
| *“Inadequate understanding of ORT and consequent limited enthusiasm for the therapy were undoubtedly major factors underlying this usage pattern.” [28]* | 1988 | Pakistan | ORS |  |
| *“Surprisingly, even among mothers aware of ORT, a third of them didnot practice it. When questioned in depth, they described various reasons including vomiting and not being advised by a medical practitioner as their reason for nonimplementation. Some considered, that ORT would worsen the diarrhoea and others had certain inherent prejudices against the use of ORT in diarrhoea.” [35]* | 1995 | India | ORS |  |
| *“It was interesting to observe that 15 (23.1~) mothers did not have any faith on ORT though they themselves had never used it. Two (3. I~) mothers who lost faith on ORT after using it, were expecting immediate stoppage of diarrhea after starting ORT but were disappointed.” [36]* | 1985 | India | ORS |  |
| *“For instance, although many mothers viewed diarrhoea as serious, they did not respond by treating with ORS. They lacked sufficient under- standing to draw the association between the risks of diarrhoea and the importance of ORS use.” [37]* | 2007 | Indonesia | ORS |  |
| **Low ORT preparation knowledge** | | | | Knowledge and Beliefs about the Intervention (Characteristics of Individuals) |
| *“some people describe SSS as ineffective, because "the preparation is not made correctly" and "some fail to understand that SSS only replaces the lost water of the body" [33]* | 1997 | Nigeria | RHF |  |
| *“Knowledge of the existence of ORT (88- 94%) and the wide use of ORT in 78- 90% of diarrhoea cases do not guarantee its correct use.” [38]* | 2005 | South Africa | RHF |  |
| *“Knowledge of the existence of ORT (88- 94%) and the wide use of ORT in 78- 90% of diarrhoea cases do not guarantee its correct use.” [38]* | 2005 | South Africa | RHF |  |
| **Low supply of ORT** | | | | Available Resources (Inner Setting) |
| *“The major source of ORS stock was ASHAs (Accredited Social Health Activists) and local rural medical practitioners (RMPs). The ORS was not available in local markets and the mothers had to be dependent on ASHAs at the time of need.” [26]* | 2017 | India | ORS |  |
| *“However, the scale-up efforts had limited success in the rural areas as there was no commercial production of ORS packets resulting in limited supplies” [23]* | 2019 | Bangladesh | ORS |  |
| *“The study also looked at issues of availability of materials at ORT corners and stockouts and found that supply of salt, sugar, ORS packages and zinc tablets in both states was insufficient, a situation that needs to be addressed urgently. Overall, ORT corner providers identified a lack of supplies as the major barrier for providing services to the users.” [24]* | 2015 | Nigeria | ORT |  |
| *“Although ORS production was sufficient, there were problems in its distribution, which led to its underuse. ORS was unavailable in some areas and in excess supply in others.” [29]* | 2002 | Philippines | ORS |  |
| *“One third to one half of these deaths could have been prevented with an effective ORT programme; yet the main obstacle to the implementation of such a programme was the lack of ORS” [25]* | 1985 | Egypt | ORS |  |
| **Low access to ORT** | | | | Available Resources (Inner Setting) |
| *“Almost half of all respondents (48%) reported having used packaged or homemade ORS in the past, and almost twice as many urban residents (62%) as rural residents (34%) had ever used ORT by any method (Table 1).... This is partly explained by the fact that packets were the preferred choice of method for both groups, yet their supply was limited in the rural setting (health workers’ homes only). Therefore, access to both supplies and information limits the potential for ORT use in rural communities.” [27]* | 1988 | Haiti | ORT |  |
| **Discouragement from health providers** | | | | Access to Knowledge and Information (Inner Setting) |
| *“The reasons for not giving ORT to diarrhoea patients were investigated. It appears that a major reason was discouragement by health providers (qualified and unqualified practitioners).” [54]* | 1997 | Bangladesh | ORS |  |
| *“Clearly not enough time was spent informing pharmacists about the importance of benefits from ORT. In addition it is apparent that the profit of 3 PT. per ORS package did not constitute enough of a unit monetary incentive for pharmacists and shopkeepers, when the sale of an antibiotic would bring in at least ten times that amount per unit sale” [57]* | 1986 | Egypt | ORS |  |
| **Unclear definition of a diarrheal episode** | | | | Knowledge and Beliefs about the Intervention (Characteristics of Individuals) |
| *“The evaluation showed that the overall usage rates of LGS were low, but this can partly be explained by the programme definition used by BRAC for a diarrhoea] episode, which is one or more watery stools per day. The effect of using this definition might be to include in the denominator a higher proportion of mild diarrhoeas, which would be unlikely to receive any form of treatment.” [46]* | 1988 | Bangladesh | RHF |  |
| **Alternative treatments preferred** | | | | Knowledge and Beliefs about the Intervention (Characteristics of Individuals) |
| *“The commonest treatment for diarrhea involved the use of drugs and allopathic medicines and the second most popular was boneji, a traditional treatment based on local herbs. Lobon-gur was third in popularity and was used in only 4-10% of diarrhea episodes. This largely explains the much higher usage in episodes of severe watery diarrhea, as found in the subsequent community survey.” [39]* | 1988 | Bangladesh | RHF |  |
| *“Another finding with important implications for ORT programs was that certain diarrheas were classified as signs of folk illnesses requiring traditional folk treatment rather than fluid replacement or other biomedical therapy.” [28]* | 1988 | Pakistan | ORS |  |
| **Relative high cost of ORT** | | | | Cost (Intervention Characteristics) |
| *“Therefore, despite the moderate cost of commercial packets, compared to other types of medical care and essential drugs, ability to pay may still serve as a barrier to utilization in a poor population, particularly when a cheaper alternative is available.” [27]* | 1988 | Haiti | ORT |  |
| *“In addition, even though the women said that ORS packets were either supplied free (at clinics) or could be purchased for about 3 rupees each (at shops), it is possible that the cost of the packets predisposed to underuse, given the cost and difficulty of transport and the general shortage of cash in this rural population.” [28]* | 1988 | Pakistan | ORS |  |
| **Low literacy of mothers** | | | | Other Personal Attributes (Characteristics of individuals) |
| *“The fact that literacy correlates with knowledge and early initiation of ORT, independent of place of residence, underscores the well-established finding that the better educated are more receptive to innovations.” [27]* | 1988 | Haiti | ORT |  |
| **Poor taste of ORT** | | | | Design and Quality and Packaging (Intervention Characteristics) |
| *“Mothers say that their children refuse to drink the solution as its taste is unpleasant.” [40]* | 1984 | Papua New Guinea | ORS |  |
| *“Of those who did not use WHO ORS, the majority said that it was because of the taste.” [45]* | 1994 | Nicaragua | ORS |  |
| **Poor acceptance of ORT** | | | | Knowledge and Beliefs about the Intervention (Characteristics of Individuals) |
| *“But an increasing majority of women express dissatisfaction with the treatment. Of the 68 women interviewed in depth, 63% expressed reservations about ORT.” [40]* | 1984 | Papua New Guinea | ORS |  |
| *“among 190 respondents who at least once failed to give ORS after a diarrheal episode, the underlying reasons for not doing so were; the child did not like it (52.6 %), thinking that ORS does not help the child much (20.0 %), forgetfulness (12.6 %), underestimating the seriousness of the disease (10.5 %) and giving preference to homemade fluids (7.9 %).” [41]* | 2016 | Ethiopia | ORS |  |
| *“Whilst WHO-ORS was cheaper for public hospitals to obtain, the ambivalent status of ORS as a non-exclusive, low-technology, inexpensive medicine, and the fact that infants were unable or unwilling to swallow the salty-tasting liquid, meant it was not well accepted by either prescribers or caretakers.” [42]* | 2003 | Thailand | ORS |  |
| *“This shows that many mothers in spite of being aware of ORS were reluctant to use ORS. The wrong perceptions of mothers about the cause of diarrhea have also led to decreased ORS use in spite of knowledge about ORS.” [43]* | 2017 | Pakistan | ORS |  |
| *“Many factors including time constraints imposed by competing responsibilities in the home and mothers questioning the credibility of oral rehydration therapy mitigate against compliance.” [44]* | 1994 | South Africa | ORS |  |
| **Low maternal knowledge of diarrhea** | | | | Knowledge and Beliefs about the Intervention (Characteristics of Individuals) |
| *“Less frequent use of the ORS packet was associated with inaccurate definitions of diarrhea, treating diarrhea by other methods than taking their child to the Centro de Salud, and not knowing ORS is used for rehydration.” [45]* | 1994 | Nicaragua | ORS |  |
| **Production and distribution costs** | | | | Cost (Intervention Characteristics) |
| *“In the context of primary health care, the dual challenges of packet production and distribution costs, as well as the need for more effective education and training of health workers and the community, remain formidable barriers to the acceptance and use of C-ORS.” [32]* | 1991 | Bangladesh | RHF |  |
| **Poor education/ training of workers** | | | | Access to Knowledge and Information (Inner Setting) |
| *“In the context of primary health care, the dual challenges of packet production and distribution costs, as well as the need for more effective education and training of health workers and the community, remain formidable barriers to the acceptance and use of C-ORS.” [32]* | 1991 | Bangladesh | RHF |  |
| *“In spite of repeated training of health workers to educate mothers to use ORT earliest in an episode of diarrhea and in adequate amounts, the grass-root level health workers failed to do so even amongst the small proportion of mothers (12.4%) educated by them. As a result, none of the mothers used ORT early and in adequate amount.” [59]* | 1991 | India | ORT |  |
| **Females not given ORT** | | | | Other Personal Attributes (Characteristics of Individuals) |
| *“The higher proportion of deaths amongst females can be explained by the lower number of females given ORS. This is related to cultural factors which resulted in the neglect of female children.” [74]* | 1987 | India | ORS |  |
| **Drugs prescribed over ORT** | | | | Access to Knowledge and Information (Inner Setting) |
| *“It is important to note that, contrary to the therapeutic practices taught during the training programme, the physicians prescribed anti-microbials to 45% and kaolin-pectin to 40% of patients. Although ORS was also prescribed along with the drugs, it was frequently the last item on the prescription and sometimes the least emphasized” [55]* | 1989 | India | ORS |  |
| *“ORS is used in 73% of episodes in children taken to a government health facility, and ORS alone, generally the most appropriate treatment, is used in about half of these cases. If, however, the child is taken to a private physician, ORS is used in only 59% of cases” [56]* | 1995 | Egypt | ORS |  |
| *“The opposition to the use of ORT alone in the treatment of acute diarrhea in children by 54.4% of pharmacists shows disregard to the guideline stipulated by WHO. It was noted” [58]* | 2014 | Nigeria | ORS |  |
| *“It has even been stated that the medical profession is the major constraint to the widespread use of ORT due to the lack of confidence in its efficacy, especially among private practitioners. High use rates of antibiotics and spasmolytics, 58% and 41% respectively, were also recorded in this study.” [53]* | 1996 | Nigeria | ORT |  |
| **Inadequate ORT program promotion** | | | | Access to Knowledge and Information (Inner Setting) |
| *“A partial explanation for the limited use of oral rehydration therapy may be that programs that promote this form of therapy have largely neglected to address the problem of bloody or mucoid diarrhea.” [51]* | 1991 | Bangladesh | ORS |  |
