# Supplementary material for: Barriers and facilitators to implementation of oral rehydration therapy in low- and middle-income countries: A systematic review
Source: PLoS One. 2021 Apr 22;16(4):e0249638. doi: 10.1371/journal.pone.0249638 (PMC8062013; doi:10.1371/journal.pone.0249638)
Supplement: S4 Table — (DOCX) [file pone.0249638.s006.docx]

**S4 Table. Facilitators to ORT Implementation**

| **Facilitator** | **Year** | **Geography** | **ORT/ORS/RHF** | **CFIR Framework** |
| --- | --- | --- | --- | --- |
| **Implementing social franchising programs** | | | | Available Resources (Inner Setting) |
| *“There was a significant difference in the use of ORS plus zinc supplementation after implementation of the social franchising program, with 13.7% of diarrhea cases treated in the intervention villages compared with 1.8% in the control villages (p < 0.001, Fig. 2).There was a significant difference in the use of ORS plus zinc supplementation after implementation of the social franchising program, with 13.7% of diarrhea cases treated in the intervention villages compared with 1.8% in the control villages (p < 0.001, Fig. 2).” [31]* | 2014 | Myanmar | ORS |  |
| **Training/supplying health workers** | | | | Access to Knowledge and Information (Inner Setting) |
| *“Our trial provides evidence that a social franchising program training and supplying local health workers results in an increase in the treatment of childhood diarrhea with ORS plus zinc supplementation in rural Myanmar.” [31]* | 2014 | Myanmar | ORS |  |
| *“the prescription of ORT for children younger than 5 years old in- creased from 31.4% to 59.4%.” [47]* | 1994 | Mexico | ORS |  |
| *“The refresher training of paediatric staff at KCH significantly altered treatment practices for children admitted with diarrhoeal diseases to the paediatric ward. During a 2-year post-training period, the use of intravenous solution to rehydrate children with diarrhoeal diseases decreased by 56%, the exclusive use of ORS for mild or moderate dehydration increased threefold” [48]* | 1990 | Malawi | ORS |  |
| **Maternal/caregiver education programs** | | | | Access to Knowledge and Information (Inner Setting) |
| *“The point of interest however is that following discontinuation of ORS packets, there was no decline in the use of ORT when mothers were educated to prepare a substitute solution with the household ingredients. Education on diarrhoeal management during the initial phase is likely to have improved the motivation among mothers to use ORT during sugar salt period.” [49]* | 1988 | India | RHF |  |
| *“In the 1960s infants arrived dehydrated, in shock, and often with hypernatraemia. In the 1980s most infants present in a reasonable state of hydration. In Tonga this change occurred suddenly in the last quarter of 1981 after a major epidemic and coincided with the health education unit teaching mothers about oral rehydration.” [60]* | 1985 | Kingdom of Tonga | ORS |  |
| *“In conclusion, with the right approach, mothers will retain what physicians and nurses teach and spread the word about rehydration therapy.” [50]* | 1986 | Egypt | ORS |  |
| **Program promoting ORT use** | | | | Engaging (Process) |
| *“Our finding that 80% of caregivers gave ORT is therefore very encouraging. The improvement probably reflects the success of the IMCI programme and SSS promotion implemented after that study” [61]* | 2013 | South Africa | RHF |  |
| **Engaging private/public sector** | | | | Cosmopolitanism (Outer Setting) |
| *“The engagement of private providers in the program was an important factor in achieving high rates of intervention compliance.“ [68]* | 2008 | India | ORS |  |
| *“Finally, even though SHOPS primarily focused the provider training component of its interventions on the private sector, caregivers obtained zinc and ORS from both private- and public sector providers. Thus, engaging both the private and public sectors will be essential to ensure increased access to ORS and zinc” [69]* | 2016 | Ghana | ORS |  |
| **Combined NGO/private sector/public sector stewardship** | | | | Cosmopolitanism (Outer Setting) |
| *“Involvement of the non-government organizations (NGOs) and the private sector along with public sector stewardship was instrumental in popularizing ORS.” [23]* | 2019 | Bangladesh | ORS |  |
| **Mass communication campaigns** | | | | Engaging (Process) |
| *“This mass awareness raising effort helped substantially shift community norms from restricted feeding to feeding to the use of home-made solution for dehydration correction.” [23]* | 2019 | Bangladesh | ORS |  |
| *“The large increase in the number of mothers using ORT, as well as the increase in the consumption of ORS that was observed during the programme (corresponding to stage II of the analysis), was supported by an extended use of the mass media for educational purposes.” [62]* | 1996 | Mexico | ORS |  |
| *“The evaluation analysis showed that greater exposure to the social marketing campaign was associated with increased use of ORASEL and with improvements in perceived availability, knowledge of the signs of diarrhea and dehydration, social support, and self-efficacy” [63]* | 2011 | Burundi | ORS |  |
| *“Regarding availability, those who felt that ORASEL was sold at an affordable price were 1.8 times as likely to be ORASEL users (p < .01), and those who felt that the scarcity of ORASEL was a barrier to use were half as likely to be ORASEL users (p < .001). Perceived availability, an opportunity variable, improved significantly with exposure to the program.” [63]* | 2011 | Burundi | ORS |  |
| *“The ‘ever administered ORS’ increased from* *18 to 54% in the experimental community and 9 to 37% in the control community. Comparing several recent ORT intervention projects, the paper concludes that a combination of a commercial approach and mass communication techniques can further ORS use.” [64]* | 1990 | Kenya | ORS |  |
| *“The different results found in Uttar Pradesh compared to Gujarat suggest that for a mass media campaign to be effective, the mass media campaign should be designed for the local context and requires consistent messaging at a high frequency.” [65]* | 2019 | India | ORS |  |
| *“The educational messages in the public service announcements were largely successful, though more in improving knowledge than practice.” [56]* | 1995 | Egypt | ORS |  |
| *“After 1985, nearly 100% of mothers knew about ORS and dehydration. Remarkably, by 1988 nearly 90% of randomly sampled mothers could mix ORS correctly on the spot.” [67]* | 1995 | Egypt | ORS |  |
| **Utilizing locals** | | | | Adaptability (Intervention Characteristics) |
| *“Use of local volunteers in delivering ORT had many-fold advantages. Since no remuneration was required to be paid, this system proved to be economic. Being a local person he/she was better accepted by the people. A village based delivery system, which is absolutely necessary to cut down mortality and hospital referrals, was thus found to be feasible.” [36]* | 1985 | India | ORS |  |
| *“Utilisation of local Village Headmen and their people in dissemination of the knowledge had two-fold advantages. Firstly, being the representatives of the local people their advice attracted better community acceptance and participation. Secondly, this minimised the health educational responsibilities of the volunteers.” [36]* | 1985 | India | ORS |  |
| *“Specifically, over the study period the healers significantly increased the mothers’ awareness, use, and proper preparation of ORS; reduced dangerous withholding of food; promoted continued feeding, including breast-feeding, during diarrhea; and reduced the use of costly commercial ORS and non-indicated drugs.” [77]* | 1988 | Brazil | RHF |  |
| **Government funding/subsidized resources** | | | | External Policies and Incentives (Outer Setting) |
| *“Since 1983 the NCDDP has guaranteed distribution of ORS to nearly all 32 000 MOH and university clinics and hospitals and 6000 private pharmacies; has trained more than 40 000 primary health care physicians, nurses, pharmacists, and others in the principles of ORT. This greatly increased the availability and accessibility of ORS. ORS usage increased, as before this Government Led program, antibiotics and drugs were the main treatment for diarrhea.” [70]* | 1990 | Egypt | ORS |  |
| *“The strategies adopted under the program have resulted in increased utilization of public health services and use of zinc and ORS for childhood diarrhea treatment.” [30]* | 2015 | India | ORS |  |
| *“Use of ORS and combined ORS and zinc for treatment of diarrhea in children under five significantly increased in focal states during the program period” [66]* | 2019 | Nigeria | ORS |  |
| *“National and statewide ORS and zinc coverage in program areas increased during the program period, and rates of coverage increases were greater than those in comparable regions. The concurrent changes in these indicators were consistent with the program’s theory of change which expected that improvements in product availability, pricing, provider dispensing, consumer demand and policy would likely contribute to overall improvements in population-level coverage rates.” [72]* | 2019 | India, Kenya, Nigeria, Uganda | ORS |  |
| **Pre-packaging ORT/zinc** | | | | Design and Quality and Packaging (Intervention Characteristics) |
| *“In the current study, the better adherence observed in the ‘central’ and ‘HC level’ arms and the absence of sizable difference between the other two arms demonstrate that the benefit is predominantly due to the effect of instructional messages provided on the pouch” [41]* | 2016 | Ethiopia | ORS |  |
| *“The findings suggested that the utilization of ORS and Zinc was significantly high in intervention areas and both of these interventions found to be concomitantly working well and it was also observed the optimal treatment with Diarrhea Pack found to be associated with low utilization of antibiotics and intravenous fluids, these findings are consistent with the findings of other studies [19,21,22].” [73]* | 2013 | Pakistan | ORS |  |
| *“Our evaluation suggests that the DPCC activities in the public sector, particularly the switch to co-packaged ORS and zinc and the widespread dissemination of this information to NMS, district health offices, and facilities, likely played an important role in the increase of both ORS and zinc. In 2016, 77% of patients with diarrhoea episodes seeking care from public sources were receiving ORS and 53% were receiving both ORS and zinc.” [71]* | 2019 | Uganda | ORS |  |
| **Health center provided education** | | | | Access to Knowledge and Information (Inner Setting) |
| *“Those who treated diarrhea by taking their child to the clinic were more likely to administer the oral solutions to their child than those who did not use the clinic. These findings suggest a role of the public health system in teaching and supporting the use of ORT.” [45]* | 1994 | Nicaragua | ORS |  |
| **Homemade ORT training** | | | | Access to Knowledge and Information (Inner Setting) |
| *“Our study demonstrates increased knowledge of ORT and ZS among community members trained by the study promoters. Training on homemade ORT increased both accessibility and affordability for community members who otherwise would have to undertake a trip to a government health facility or pay for ORT in a pharmacy or shop.” [52]* | 2017 | Guatemala | RHF |  |
| **Acceptance of Home-made ORT** | | | | Adaptability (Intervention Characteristics) |
| *“Compliance after 48 hours of treatment was found to be considerably better among caretakers providing the HC-ORT. This may in part be because of its similarity to a locally prepared cereal, known as buluka (Oromo) or atemeet (Amhara), thus enhancing its cultural acceptability.” [75]* | 1994 | Ethiopia | RHF |  |
| **Availability of ORT** | | | | Available Resources (Inner Setting) |
| *“Regular availability of zinc and ORS in the public sector is critical for the treatment of diarrhea cases with both zinc and ORS” [30]* | 2015 | India | ORS |  |
| **Focal district interventions** | | | | Engaging (Process) |
| *“Our results demonstrate that focal district interventions were significantly associated with increased use of ORS to treat diarrhea in children under five.” [65]* | 2019 | India | ORS |  |
| **Rice-based ORT as an alternative** | | | | Adaptability (Intervention Characteristics) |
| *“Thus, the most effective home- made solutions so far evolved for ORT through careful studies are similar to the food preparations traditional in Bangladeshi households. It was not surprising, therefore, to find prompt general acceptance and utilisation of the rice-salt OR solution as described in this study.” [76]* | 1985 | Bangladesh | RHF |  |
